# Tissue factor as a potential coagulative/vascular marker in relapsing-remitting multiple sclerosis

**DOI:** 10.3389/fimmu.2023.1226616

**Published:** 2023-07-31

**Authors:** Tatiana Koudriavtseva, Svetlana Lorenzano, Maria Cellerino, Mauro Truglio, Marco Fiorelli, Caterina Lapucci, Giovanna D’Agosto, Laura Conti, Annunziata Stefanile, Silvana Zannino, Maria Maddalena Filippi, Antonio Cortese, Carlo Piantadosi, Marta Maschio, Andrea Maialetti, Edvina Galiè, Marco Salvetti, Matilde Inglese

**Affiliations:** ^1^ Medical Direction, Istituto di Ricovero e Cura a Carattere Scientifico (IRCCS) Regina Elena National Cancer Institute, Rome, Italy; ^2^ Department of Clinical Experimental Oncology, Istituto di Ricovero e Cura a Carattere Scientifico (IRCCS) Regina Elena National Cancer Institute, Istituti Fisioterapici Ospitalieri (IFO), Rome, Italy; ^3^ Department of Human Neurosciences, Sapienza University of Rome, Rome, Italy; ^4^ Department of Neuroscience, Rehabilitation, Ophthalmology, Genetics, Maternal and Child Health (DINOGMI), University of Genoa, Genoa, Italy; ^5^ Clinical Pathology and Cancer Biobank, Istituto di Ricovero e Cura a Carattere Scientifico (IRCCS) Regina Elena National Cancer Institute, Rome, Italy; ^6^ Clinical Pathology and Microbiology, Istituto di Ricovero e Cura a Carattere Scientifico (IRCCS) San Gallicano Dermatological Institute, Rome, Italy; ^7^ Neuroscience and Imaging, Fatebenefratelli Hospital, Rome, Italy; ^8^ Unità Operativa Complessa (UOC) Neurology, San Giovanni-Addolorata Hospital, Rome, Italy; ^9^ Department of Neuroscience Mental Health and Sensory Organs (NEMOS), Sapienza University, Rome, Italy; ^10^ Istituto di Ricovero e Cura a Carattere Scientifico (IRCCS) Istituto Neurologico Mediterraneo Neuromed, Pozzilli, Italy; ^11^ Department of Neurology, Mount Sinai Hospital, New York, NY, United States

**Keywords:** multiple sclerosis, relapse, tissue factor, biomarker, coagulation

## Abstract

**Objectives:**

Recent studies supported coagulation involvement in multiple sclerosis, an inflammatory-demyelinating and degenerative disease of the central nervous system. The main objectives of this observational study were to identify the most specific pro-coagulative/vascular factors for multiple sclerosis pathogenesis and to correlate them with brain hemodynamic abnormalities.

**Methods:**

We compared i) serum/plasma levels of complement(C)/coagulation/vascular factors, viral/microbiological assays, fat-soluble vitamins and lymphocyte count among people with multiple sclerosis sampled in a clinical remission (*n*=30; 23F/7M, 40 ± 8.14 years) or a relapse (*n*=30; 24F/6M, age 41 ± 10.74 years) and age/sex-matched controls (*n*=30; 23F/7M, 40 ± 8.38 years); ii) brain hemodynamic metrics at dynamic susceptibility contrast-enhanced 3T-MRI during relapse and remission, and iii) laboratory data with MRI perfusion metrics and clinical features of people with multiple sclerosis. Two models by Partial Least Squares Discriminant Analysis were performed using two groups as input: (1) multiple sclerosis vs. controls, and (2) relapsing vs. remitting multiple sclerosis.

**Results:**

Compared to controls, multiple sclerosis patients had a higher Body-Mass-Index, Protein-C and activated-C9; and a lower activated-C4. Levels of Tissue-Factor, Tie-2 and P-Selectin/CD62P were lower in relapse compared to remission and HC, whereas Angiopoietin-I was higher in relapsing vs. remitting multiple sclerosis. A lower number of total lymphocytes was found in relapsing multiple sclerosis vs. remitting multiple sclerosis and controls. Cerebral-Blood-Volume was lower in normal-appearing white matter and left caudatum while Cerebral-Blood-Flow was inferior in bilateral putamen in relapsing versus remitting multiple sclerosis. The mean-transit-time of gadolinium-enhancing lesions negatively correlated with Tissue-Factor. The top-5 discriminating variables for model (1) were: EBV-EBNA-1 IgG, Body-Mass-Index, Protein-C, activated-C4 and Tissue-Factor whereas for model (2) were: Tissue-Factor, Angiopoietin-I, MCHC, Vitamin A and T-CD3.

**Conclusion:**

Tissue-factor was one of the top-5 variables in the models discriminating either multiple sclerosis from controls or multiple sclerosis relapse from remission and correlated with mean-transit-time of gadolinium-enhancing lesions. Tissue-factor appears a promising pro-coagulative/vascular biomarker and a possible therapeutic target in relapsing-remitting multiple sclerosis.

**Clinical trial registration:**

ClinicalTrials.gov, identifier NCT04380220.

## 1 Introduction

Multiple sclerosis is a chronic, immune-mediated disease characterized by the interaction of inflammatory, demyelinating, and degenerative processes within the central nervous system (CNS) (1). Current concepts on the pathogenesis of multiple sclerosis indicate that B cells and innate immunity, together with T cells, play a crucial role in disease progression (1, 2). In particular, innate immunity appears to stimulate and modulate adaptive immunity throughout the disease course (2). The primary effector processes of innate immunity are considered inflammation and coagulation (3). Understanding the close relationship between these two phenomena is key to comprehending the “immune-thrombotic” process, which can be induced by direct and functional vascular injury, such as that caused by hypoxia, sepsis, malignancy, and inflammation (4). Interestingly, microvascular abnormalities within the CNS have been suggested as contributing factors to multiple sclerosis pathogenesis (5). In our previous work (6), we described in detail the possible role of coagulation/complement and platelet activation in the context of multiple sclerosis activity. Briefly, endothelial cell dysfunction, increased coagulation activity during disease exacerbation, excessive platelet activation, evidence of oxidative stress, perivascular fibrin(ogen) deposition, higher plasma levels of prothrombin and factor X (FX), complement activation, vascular occlusion within demyelinating lesions, altered blood-brain barrier (BBB) permeability, and hypoxia-like tissue injury have all been reported in people with multiple sclerosis (6, 7).

In recent years, various MRI techniques have been developed to assess cerebral perfusion in multiple sclerosis, including dynamic-susceptibility contrast-enhanced (DSC) perfusion MRI (5, 8, 9). Using this technique, many cross-sectional studies have identified altered perfusion parameters with different combinations of either reduced cerebral blood flow (CBF) or reduced cerebral blood volume (CBV) and elevated mean transit time (MTT) in patients with multiple sclerosis compared with controls in both normal-appearing white matter (NAWM) and normal-appearing gray matter (NAGM) (5, 8, 9). It is conceivable that the global hypoperfusion in GM and WM of multiple sclerosis patients may be determined by the overall deceleration of blood flow due to the inflammatory-thrombotic processes (8).

As already reported in our previous work describing this study protocol (7), among the causes of neuroinflammation, there are recurrent and chronic infections accompanied by local innate immunity activation and consequent adaptive immune response, which in turn induces immune-thrombotic events. Indeed, coagulation is activated during viral infections and plays multiple functions in the host immune system (10). Several studies have suggested a viral etiology of multiple sclerosis and diverse viruses have been proposed as potential triggering agents (11).

Altogether, this evidence has pointed out the strong interplay between coagulation and inflammation with infections in the pathophysiology of multiple sclerosis, as well as its association with brain perfusion metrics, thus suggesting possible therapeutic targets that may integrate existing treatments. The primary objectives of the present study were: i) to identify the most specific pro-coagulative/vascular factors that, along with their neuroimaging functional correlates in terms of brain hemodynamic abnormalities, could play a role in the pathogenesis of multiple sclerosis and its different phases (relapsing and remitting); ii) to investigate the possible association of coagulation and hemodynamic alterations with multiple sclerosis clinical outcomes and infectious markers (7).

## 2 Materials and methods

### 2.1 Study population and design

**Figure 1:**
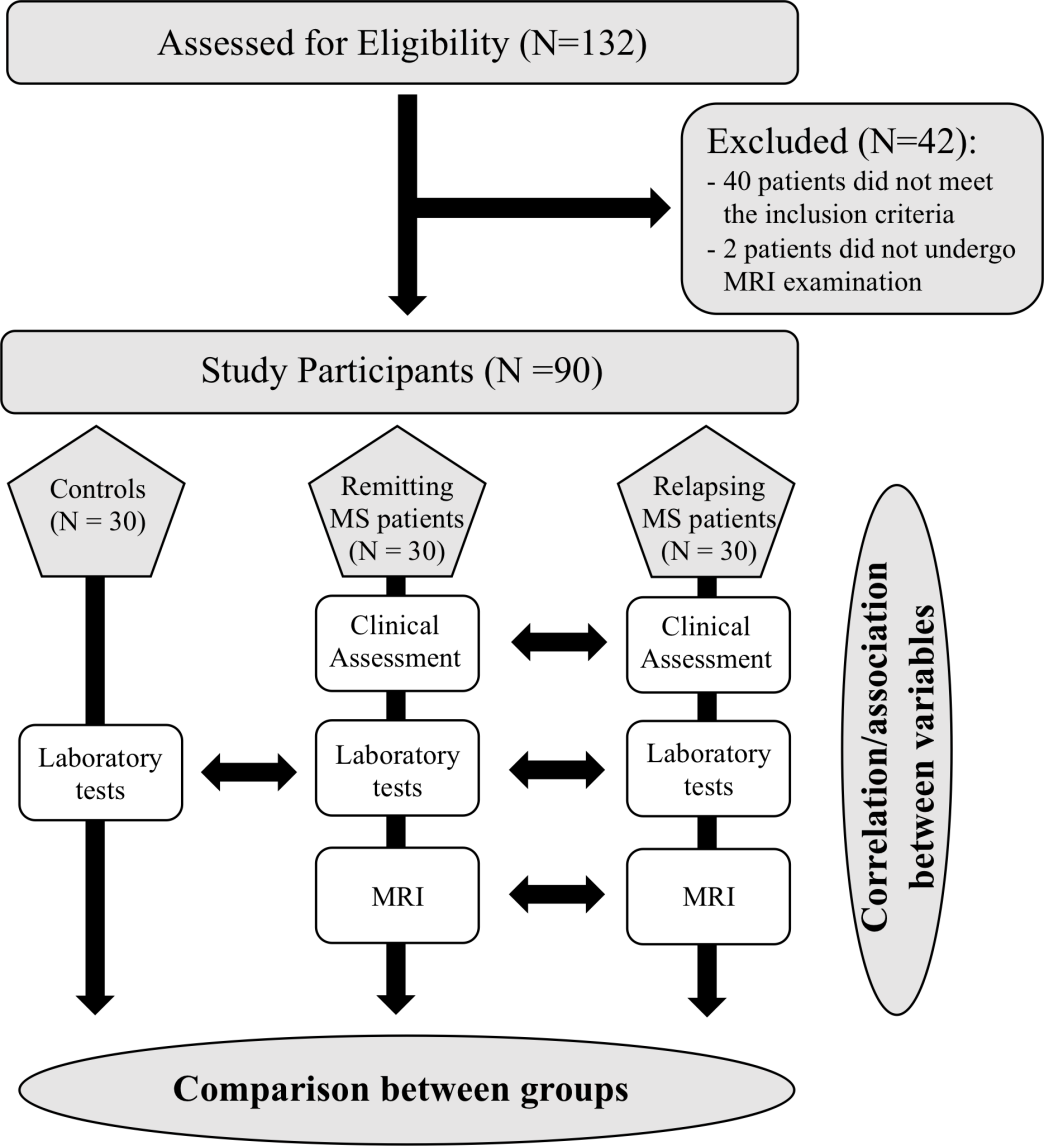
Flow chart of the study.

The study protocol has been already described in detail in a previous publication (**Figure 1** flow chart) (7); the ClinicalTrials.gov Identifier is NCT04380220. The study protocol and statistical analysis plan are available in the **Supplementary Material**. Briefly, this is a multi-center, prospective, observational, controlled study conducted in two centers (the Multiple Sclerosis Center of the Sapienza University of Rome and the Multiple Sclerosis Center of the IRCCS Regina Elena National Cancer Institute) from 6 September 2017 to 3 May 2021.

Individuals with multiple sclerosis were included if they fulfilled the following inclusion criteria: age 18–60 years; relapsing-remitting multiple sclerosis diagnosis according to the revised McDonald’s criteria (12); being untreated or treated only with “first-line” immunomodulatory therapies (i.e., interferon, glatiramer acetate, teriflunomide, dimethyl-fumarate). Exclusion criteria were: pregnancy; co-existing neoplastic, hematologic, thyroid, metabolic, thrombotic, or autoimmune diseases; drug or alcohol addiction; therapy with immunosuppressive drugs, steroids, or any medication interfering with coagulation. Patients were defined as “relapsing” if, at the time of enrollment, they had ongoing symptoms suggesting a relapse (defined as a manifestation of new or worsened neurological symptoms lasting for more than 24 hours) (1, 12) and “remitting” if no relapses occurred in the previous 2 months. Relapsing multiple sclerosis patients had to be enrolled before starting steroid therapy.

Controls matched by age and sex with remitting patients were also included in the study. They were recruited among the personnel of the participating centers and were not affected by disease, did not undergo recent surgery procedures, or did not have recent trauma/injuries, based on their clinical history, life habits, and the standard medical checkups routinely performed for their work.

For each patient, at enrolment, data on demographics and clinical history and peripheral blood sample were collected and the assessment of physical disability using the Expanded Disability Status Scale (EDSS) (13) and Multiple Sclerosis Functional Composite (MSFC) (14) was carried out. DSC perfusion 3.0-T MRI (7) was performed within two weeks of enrolment. For the controls, only demographic data and laboratory markers were collected.

All enrolled subjects provided their signed informed consent prior to their inclusion in the study. The subjects’ consent was obtained according to the Declaration of Helsinki.

The study, including the informed consent form, was approved by the Ethics Committees of the IRCCS Regina Elena National Cancer Institute and of the Sapienza University of Rome.

### 2.2 Study endpoints

The primary endpoint of our study was the determination of potential differences in terms of serum/plasma concentrations of complement and pro-coagulative/vascular markers among the participants’ groups. Key secondary endpoints included: i) the assessment of the relative CBF, CBV and MTT, as well as of the number and volume of enhancing lesions and the comparison of these parameters between relapsing- and remitting-multiple sclerosis patients; ii) to explore the relationships between demographic/clinical features and laboratory data and MRI perfusion findings in the patient groups. Additional secondary endpoints involved: i) the evaluation of the complete blood cell count (CBC) and viral/microbiological serological assays in all participant groups; ii) the identification of potential differences in terms of lymphocyte sub-populations between relapsing- and remitting-multiple sclerosis patients. As exploratory endpoints, differences in terms of laboratory markers between male and female controls and patients were also investigated.

### 2.3 Laboratory and neuroimaging data

The interventional methods have been described in detail in a previous publication (7). The lists of the laboratory biomarkers and MRI metrics measured in the study are reported in **Supplementary Tables 1**, **2**, respectively.

### 2.4 Statistical analysis

Overall, 90 subjects (30 for each group) were enrolled in order to compare the level of complement C4a (55). By using the analysis of variance (ANOVA) test, this sample size allowed to detect effect size values [delta=(miA–miB)/sigma] equal to at least 0.71, with a statistical power of 80%, to a level of significance of 5%. In all analyses, potential confounding factors including treatment, sex, and age were controlled for.

Descriptive statistics were used to summarize pertinent study information. Correlations between quantitative variables were assessed with Spearman’s rho coefficient. The associations were analyzed by the Fisher exact test or Chi Square test for trends. Comparisons between disease subgroups and control group were carried out for different variables, using either Student’s t-test or ANOVA. If the ANOVA showed a statistical difference between subgroups, a *post-hoc* analysis with Bonferroni-Holm correction for multiple comparisons was performed. For non-normally distributed data, non-parametric (Mann Whitney-U or Kruskal-Wallis-H) tests were used. In this study, missing data were identified to be Missing Completely at Random (MCAR), indicating that the missingness was not related to any measured or unmeasured variables. To deal with missing data, a pairwise deletion approach was employed when necessary. This approach removes cases with missing data pairwise, meaning that only the incomplete observations are excluded from the analyses, leaving the remaining complete data in the study. Given the MCAR assumption, this approach is an appropriate and bias-free way to handle missing data in the study. The level of significance was set at two-tailed *P* ≤ 0.05 (SPSS version 20.0, SPSS Inc., Chicago, Illinois, USA).

Partial Least Squares Discriminant Analysis (PLS-DA) was used in order to identify the variables that had a possible role in the identification of patients’ groups (15). The analysis was performed using the ‘pls’ package in R for classification and prediction of data. In order to define the variables responsible for the measured variance in PLS-DA prediction models, variable importance in projection (VIP) scores were calculated.

A random forest classifier was built by hold-out analysis using the ‘caret’ package in R, in which the model was created using the top-5 VIP variables from the PLS-DA; the model was trained on 70% of the data and tested on the remaining 30% in order to prevent overfitting.

To obtain more precise curves and assess the performance of the models on unseen data, we used a repeated 100-fold cross-validation that consisted of 100 train and test splits. The cross-validation error curves and performances were averaged. The predictive value of each variable in the random forest models was calculated by Predictive Accuracy. The model’s performance was further evaluated through the AUC on the test set using the ‘pROC’ R package.

### 2.5 Data availability

An *ad-hoc* secure database has been established to collect standardized data from centers and to allow adequate data storage and sensitive data protection. Raw anonymized data will be made available upon reasonable request from any qualified investigator to the corresponding author.

## 3 Results

### 3.1 Population demographic and clinical characteristics

Overall, 132 patients were screened; of these, 40 patients did not meet the inclusion criteria and two other patients, although recruited, did not undergo MRI examination and were, therefore, excluded. Thirty controls were identified and matched by age and sex with the remitting patients. A total of 90 subjects (30 relapsing-multiple sclerosis, 30 remitting-multiple sclerosis and 30 controls) were enrolled in the study and included in the final analysis.

Demographic and clinical data of study groups are reported in **Table 1**. We observed a significantly higher body mass index (BMI) in both patient groups compared to controls. The mean (SD) number of total relapses, as well as relapses occurred within the previous 24 and 12 months, and baseline EDSS scores were significantly higher in relapsing-multiple sclerosis patients compared to those in remission. No differences between the two patient groups were observed in terms of the distribution of their ongoing treatments [untreated (U-), interferon (IFN-), other first line treatments (OT-)].

**Table 1:** Demographic and clinical characteristics of controls and relapsing- and remitting-multiple sclerosis patients.

	Controls (n=30)	Remitting-MS (n=30)	Relapsing-MS (n=30)	P-value*
Female; n (%)	23 (76%)	23 (76%)	24 (80%)	0.938
Age; mean (SD), y	40 (8.38)	40 (8.14)	41 (10.74)	0.920
Age at onset; mean (SD), y	–	28.8 (8.45)	29.3 (9.43)	0.807
BMI; mean (SD)	21.51 (2.78)	24.16 (4.07)	24.39 (4.25)	0.006^a^
Disease duration; mean (SD), y	–	15.56 (6.46)	15.33 (8.36)	0.900
Total relapses; n; median (IQR)	–	2 (1)	4 (3.75)	0.002
Relapses in the previous 24-months; n; median (IQR)	–	0 (0)	1 (2)	<0.001
Relapses in the previous 12-months; n; median (IQR)	–	0 (0)	1 (1)	<0.001
EDSS score at baseline; median (IQR)	–	1.5 (1.375)	3.25 (1.875)	<0.001
MSFC; mean (SD), consisted of: - 9-HPT, z score; mean (SD) - T25-FW, z score; mean (SD) - PASAT, z score; mean (SD)	–	0.06 (0.32) 0.03 (0.63) 0.26 (0.07) - 0.11 (0.53)	- 0.007 (0.44) - 0.24 (0.68) 0.26 (0.05) - 0.03 (0.80)	0.511 0.115 0.958 0.663
Therapy; n - U - IFN - OT	- - -	8 (26.7) 12 (40) 10 (33.3)	8 (26.7) 6 (20) 16 (53.3)	0.184

BMI, body mass index; EDSS, Expanded Disability Status Scale; 9-HPT, 9 Hole peg test; IFN, Interferon; IQR, interquartile range; MS, multiple sclerosis; MSFC, Multiple Sclerosis Functional Composite; OT, Other Therapy; PASAT, Paced Auditory Serial Addition Task; SD, standard deviation; T25-FW, Timed 25-Foot Walk; U, Untreated.

*Chi-square (sex, therapy), ANOVA (age, BMI), Mann-Whitney (age at onset, disease duration, total relapses, relapses previous.

24-months, relapses previous 12-months, EDSS), t-test (9-HPT, T25WT, PASAT, MSFC).

a Post-hoc analysis: controls vs relapsing-MS: p = 0.011; controls vs remitting-MS: p=0.021.

### 3.2 Differences in terms of laboratory biomarkers

Statistically significant differences between-groups in serum/plasma laboratory variables are reported in **Table 2** (all differences are reported in **Supplementary Table 3**).

**Table 2:** Statistically significant between-group differences in terms of laboratory biomarkers in controls and remitting- and relapsing-multiple sclerosis patients.

A - Coagulation/complement biomarkers	Controls (n=30)		Remitting-MS (n=30)		Relapsing-MS (n=30)		
	Mean (SD)	Median (IQR)	Mean (SD)	Median (IQR)	Mean (SD)	Median (IQR)	*P*-value^*^
PC	99.13 (14.44)	97.5 (25.25)	110.66 (18.07)	116.5 (28.5)	116.86 (20.74)	117 (30)	0.001^a^
aC4	5.78 (5.21)	4.75 (1.725)	2.75 (1.43)	2.3 (1.125)	3.91 (3.13)	2.85 (2.107)	0.001^b^
aC9	3.38 (1.13)	3.4 (1.675)	4.91 (3.03)	4.3 (3.3)	5.42 (4.13)	4.4 (3.54)	0.019^c^
AngI	9.40 (4.04)	8.38 (4.34)	7.31 (2.96)	7.26 (2.84)	10.57 (4.83)	9.76 (5.75)	0.009^d^
TF	110.85 (56.31)	120.69 (101.11)	94.06 (55.83)	99.23 (104.22)	45.03 (37.66)	22.72 (58.67)	<0.001^e^
Tie-2	49.60 (17.58)	43.15 (10.775)	46.45 (17.36)	40.85 (22.425)	34.42 (19.94)	35.7 (25.3)	0.002^f^
P-Sel	245.85 (120.48)	197 (156)	216.26 (82.15)	216.5 (131)	169.41 (173.51)	160 (215.4)	0.020^g^
VitA°	40.75 (18.15)	32.68 (21.925)	43.15 (12.77)	41.63 (12.43)	105.18 (50.07)	100.8 (101.13)	<0.001^h^
VitK°	163.89 (110.80)	126.49 (119.56)	166.75 (107.20)	148.405 (167.115)	295.51 (64.84)	272.455 (299.222)	<0.001^i^
B - Serological viral/microbiological assay	Controls (n=30)		Remitting-MS (n=30)		Relapsing-MS (n=30)		
	Mean (SD)	Median (IQR)	Mean (SD)	Median (IQR)	Mean (SD)	Median (IQR)	*P*-value^*^
EBV-EBNA IgG	26.81 (7.48)	27.65 (8.325)	31.05 (6.35)	32.15 (6.3)	31.89 (4.66)	32 (5.3)	0.014^l^
InfA IgA	2.49 (3.23)	1.45 (0.825)	2.91 (2.72)	2.1 (1.725)	1.71 (1.23)	1.35 (0.75)	0.029^n^
InfA IgG	11.59 (7.61)	8.75 (9.275)	15.87 (7.49)	15.7 (12.375)	17.88 (11.98)	12.45 (21.45)	0.041^m^
Sch IgG	4.79 (1.74)	4.6 (2.225)	4.75 (2.28)	4.15 (2.25)	3.86 (1.41)	3.4 (0.65)	0.023°
C - Complete blood cell count	Controls (n=30)		Remitting-MS (n=30)		Relapsing-MS (n=30)		
	Mean (SD)	Median (IQR)	Mean (SD)	Median (IQR)	Mean (SD)	Median (IQR)	*P*-value^*^
MCHC	33.11 (0.79)	33.2 (0.875)	32.94 (0.66)	32.85 (0.775)	33.45 (0.74)	33.2 (1.25)	0.027^p^
Lym	1.91 (0.55)	1.82 (0.607)	1.86 (0.57)	1.84 (0.8)	1.49 (0.60)	1.48 (0.968)	0.014^q^
Neu/Lym	1.85 (0.68)	1.795 (0.995)	2.24 (1.11)	2.15 (1.08)	3.42 (2.51)	2.515 (2.23)	0.005^r^
Plt/Lym	139.87 (43.82)	132.345 (63.752)	149.16 (66.38)	143.23 (80.91)	227.66 (2.2.92)	173.545 (108.295)	0.016^s^
CD3+T	–	–	1.43 (0.48)	1.406 (0.764)	1.11 (0.51)	1.024 (0.528)	0.015
CD4+T	–	–	0.92 (0.29)	0.892 (0.43475)	0.73 (0.38)	0.653 (0.322)	0.043
CD8+T	–	–	0.47 (0.25)	0.405 (0.291)	0.34 (0.18)	0.336 (0.257)	0.028
CD19+B	–	–	0.30 (0.15)	0.275 (0.17)	0.22 (0.13)	0.223 (0.128)	0.025

IQR, interquartile range; MS, multiple sclerosis; SD, standard deviation; for the abbreviations of all laboratory parameters, see **Supplementary Table 1**.

°In the context of vitamin assessment, data were available in 57 subjects for Vit A and K (22 controls, 18 remitting- and 17 relapsing- MS patients).

^*^Kruskall-Wallis or ANOVA according to the variable distribution.

Significant differences are reported in bold; statistically significant post-hoc analysis details are described below:

a P=0.042 for controls vs Remitting-MS; p=0.001 for controls vs Relapsing-MS.

b P=0.002 for controls vs Remitting-MS; p=0.008 for controls vs Relapsing-MS; p=0.039 for Relapsing-MS vs Remitting-MS.

c P=0.022 for controls vs Remitting-MS; p=0.017 for controls vs Relapsing-MS.

d P=0.005 for Relapsing-MS vs Remitting-MS.

e P<0.001 for Relapsing-MS vs controls; p<0.001 for Relapsing-MS vs Remitting-MS.

f P<0.001 for Relapsing-MS vs controls; p=0.032 for Relapsing-MS vs Remitting-MS.

g P=0.013 for Relapsing-MS vs controls; p=0.032 for Relapsing-MS vs Remitting-MS.

h P<0.001 for Relapsing-MS vs controls; p<0.001 for Relapsing-MS vs Remitting-MS.

i P<0.001 for Relapsing-MS vs controls; p<0.001 for Relapsing-MS vs Remitting-MS.

l P=0.017 for controls vs Remitting-MS; p=0.011 for controls vs Relapsing-MS.

m P=0.037 for controls vs Remitting-MS; p=0.037 for controls vs Relapsing-MS.

n P=0.014 for Relapsing-MS vs Remitting-MS.

° P=0.014 for Relapsing-MS vs controls; p=0.038for Relapsing-MS vs Remitting-MS.

p P=0.023 for Relapsing-MS vs Remitting-MS.

q P=0.020 for Relapsing-MS vs controls; p=0.047 for Relapsing-MS vs Remitting-MS.

r P=0.002 for Relapsing-MS vs controls; p=0.049 for Relapsing-MS vs Remitting-MS.

s P=0.025 for Relapsing-MS vs controls C; p=0.026 for Relapsing-MS vs Remitting-MS.

Compared to controls, patient groups exhibited: higher Protein C (PC) and activated complement 9 (aC9) with a trend towards statistical significance for higher D-dimer levels (*P*=0.07); and lower aC4 levels with a trend for lower Protein S (p=0.06) (**Table 2A**, **Supplementary Table 3**). Angiopoietin-I was higher in relapsing-multiple sclerosis patients as compared to those in remission. Furthermore, levels of Tissue Factor (TF), Tie-2 and P-Selectin/CD62P were lower in the relapsing population as compared to both remitting patients and controls. Regarding vitamins, data were only available for 57 participants (22 controls, 18 remitting- and 17 relapsing-multiple sclerosis patients) and we found higher values of vitamins A and K in relapsing patients compared to both remitting patients and controls.

In terms of viral/microbiological serological assays, EBV-EBNA-1 IgG and InfluenzaA IgG were higher in the patient groups than in controls; InfluenzaA IgA levels were lower in relapsing compared to remitting multiple sclerosis patients; and Schistosoma IgG were lower in relapsing compared to both remitting-multiple sclerosis patients and controls (**Table 2B**).

Regarding the CBC, a significantly lower number of total lymphocytes was found in relapsing multiple sclerosis patients compared to remitting multiple sclerosis patients and controls, as well as a higher MCHC in relapsing patients compared to those in remission (**Table 2C**). Interestingly, both neutrophil/lymphocyte and platelet/lymphocyte ratios were higher in relapsing patients compared to remitting multiple sclerosis patients and controls while CD3+ T-cells, CD4+ T-cells, CD8+ T-cells and CD19+ B-cells were lower in relapsing patients compared to remitting patients (**Table 2C**). When analyses were performed by treatment in the patient groups, we found a higher number of B-cells in IFN-treated remitting-multiple sclerosis patients compared to OT-treated relapsing patients (*P*=0.007) and a lower number of NK cells in INF-treated remitting patients compared to U-remitting-multiple sclerosis patients (*P*=0.004).

Subgroup analysis by sex on laboratory markers were also performed. Interestingly, we found significantly lower PT, D-dimer, aC4, anti-prothrombin IgM and anti–Annexin IgM levels in males compared to females (**Supplementary Table 4**). Conversely, males had significantly higher levels of Protein S, FII, FVII, and C3. Male participants had significantly lower platelets, RDW, and PLR values while levels of RBC, Hb, HCT and MCHC were higher in males compared to females. Analyses restricted to the patient group showed similar findings.

### 3.3 Differences in terms of MRI metrics

The mean (SD) number and volume of gadolinium-enhancing lesions were significantly higher in relapsing patients compared to those in remission [1.57 (2.54) vs 0.07 (0.26), and 0.18 (0.3) vs 0.009 (0.04) ml, respectively, *P*<0.001 for all] (**Supplementary Table 5**).

Regarding perfusion metrics, data concerning CBV, CBF and MTT were evaluated in the context of gadolinium-enhancing lesions as well as in the NAWM and in the deep NAGM of multiple sclerosis patients. Given the very low number of active lesions in remitting patients, a comparison of the perfusion metrics between patient groups was not possible.

The mean (SD) CBV of relapsing-multiple sclerosis patients was significantly lower compared to remitting-multiple sclerosis patients, either in NAWM (*P*=0.047) or the left caudatum (*P*=0.044). Similarly, a lower mean (SD) CBF in relapsing patients versus those in remission was found in both the left (*P*=0.028) and right putamen (*P*=0.037).

### 3.4 Relationships between demographic/clinical features, laboratory data, and MRI metrics

Correlations between patients’ demographic/clinical features, laboratory and MRI data with Spearman’s rho>0.5 and *P*<0.05 are shown in **Supplementary Table 6**.

When considering the relationships between demographic/clinical metrics and coagulation/vascular/complement markers, we observed: a negative correlation between EDSS and both MSFC and 9-HPT z-scores and a positive correlation between the number of relapses in the previous 12- and 24-months and vitamin A levels, and between BMI and C3 (**Table 3**). Interestingly, C3 levels positively correlated with fibrinogen and FX, while PC concentration positively correlated with both FX and FVII; FX and FVII exhibited a positive correlation with PT (**Supplementary Table 6**).

**Table 3:** Relevant statistically significant correlations between demographic, clinical and radiological (MRI) markers in the relapsing- and remitting-multiple sclerosis patients’ groups.

Variable 1	Variable 2	Spearman’s rho	*P*-value
EDSS	MSFC	-0.571	<0.0001
EDSS	9-HPT z-scores	-0.594	<0.0001
Number of relapses in the previous 24 months	Vitamin A	0.512	0.0004
Number of relapses in the previous 12 months	Vitamin A	0.540	0.0002
BMI	C3	0.592	<0.0001
Age	rCBV leakage* active (Gd+) lesions	0.704	0.0023
Disease duration	rCBF leakage* active (Gd+) lesions	0.761	0.0006
Number of relapses in the previous 12 months	rCBF leakage* active (Gd+) lesions	-0.734	0.0012
PC	rCBV leakage* active (Gd+) lesions	0.665	0.0049
TF	MTT leakage* active (Gd+) lesions	-0.746	0.0009
P-Selectin\CD62P	MTT leakage* active (Gd+) lesions	-0.645	0.007
Vit K	rCBV leakage* active (Gd+) lesions	-0.827	0.0017
Vit K	rCBF leakage* active (Gd+) lesions	-0.720	0.0125
Vit K	MTT leakage* active (Gd+) lesions	-0.70	0.0182
CMV IgM	MTT leakage* active (Gd+) lesions	0.828	0.0001
Inf B IgA	rCBV leakage* active (Gd+) lesions	0.777	0.0004

*A leakage correction analysis was also performed, in order to correct for contrast agent extravasation.

CMV IgM, Cytomegalovirus IgM; Gd+, gadolinium-enhancing lesions; Inf B IgA, Influenza B IgA; MTT, Mean Transit Time; rCBF, relative Cerebral Blood Flow; rCBV, relative Cerebral Blood Volume; TF, Coagulation Factor III/Tissue Factor; Vit K, vitamin K.

Relevant significant correlations between demographic/clinical/laboratory markers and MRI metrics emerged only for perfusion parameters of Gd-enhancing lesions (**Table 3**). Specifically, disease duration positively correlated with the CBF of active lesions, while the number of relapses in the previous 12 months and Vitamin K showed a negative correlation with this MRI metric; the CBV of Gd-enhancing lesions positively correlated with either patients’ age, PC or InfluenzaB IgA, and negatively correlated with Vitamin K. Notably, a negative correlation was found between MTT and TF, Vitamin K and P-Selectin\CD62P.

### 3.5 Data driven prediction of multiple sclerosis

**Figure 2:**
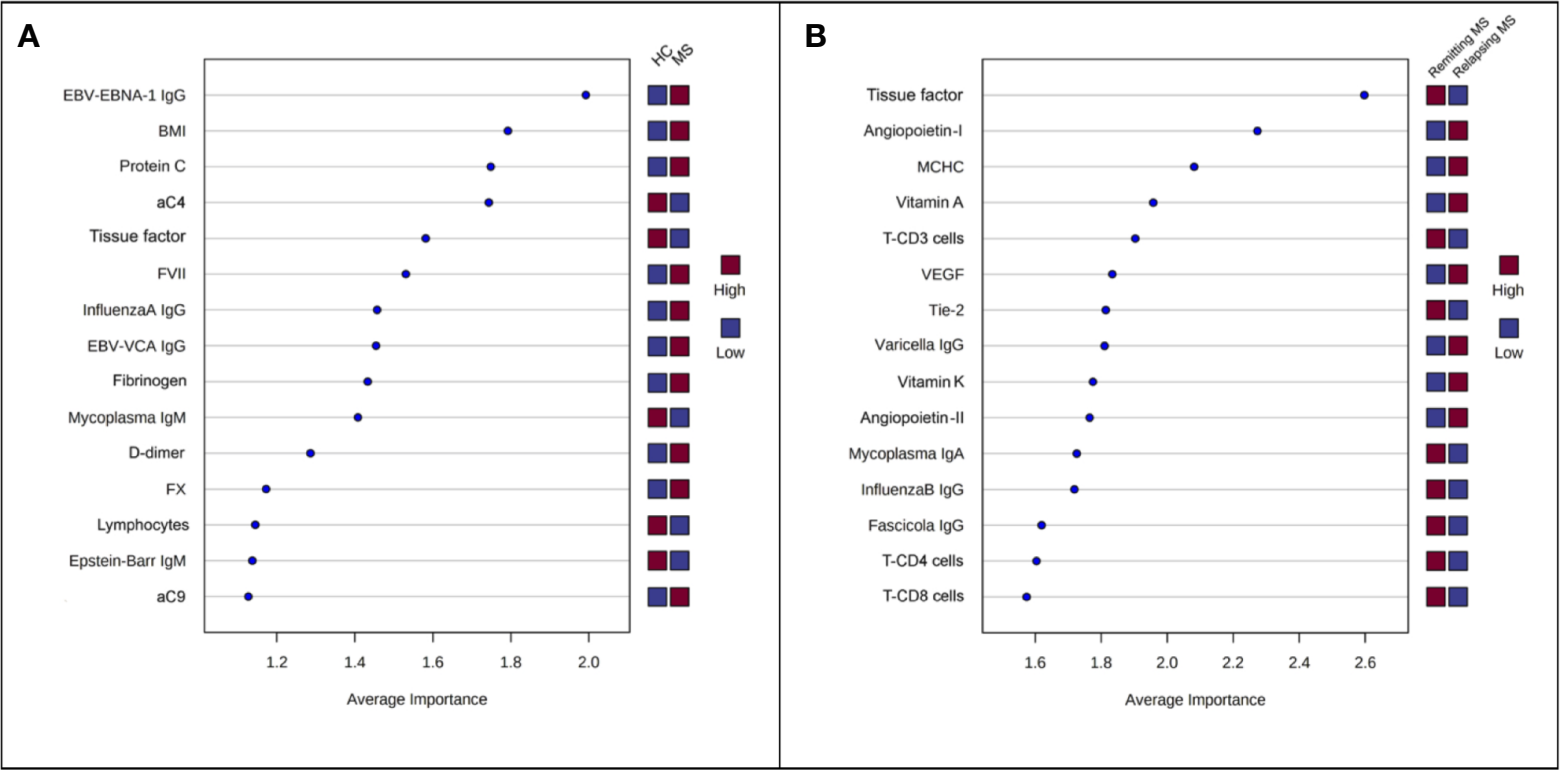
Variables ranked by Average Importance score calculated by PLS-DA. **(A)** Model (1), controls vs. multiple sclerosis (MS) (remitting + relapsing); **(B)** Model (2), remitting MS vs. relapsing MS.

**Figure 3:**
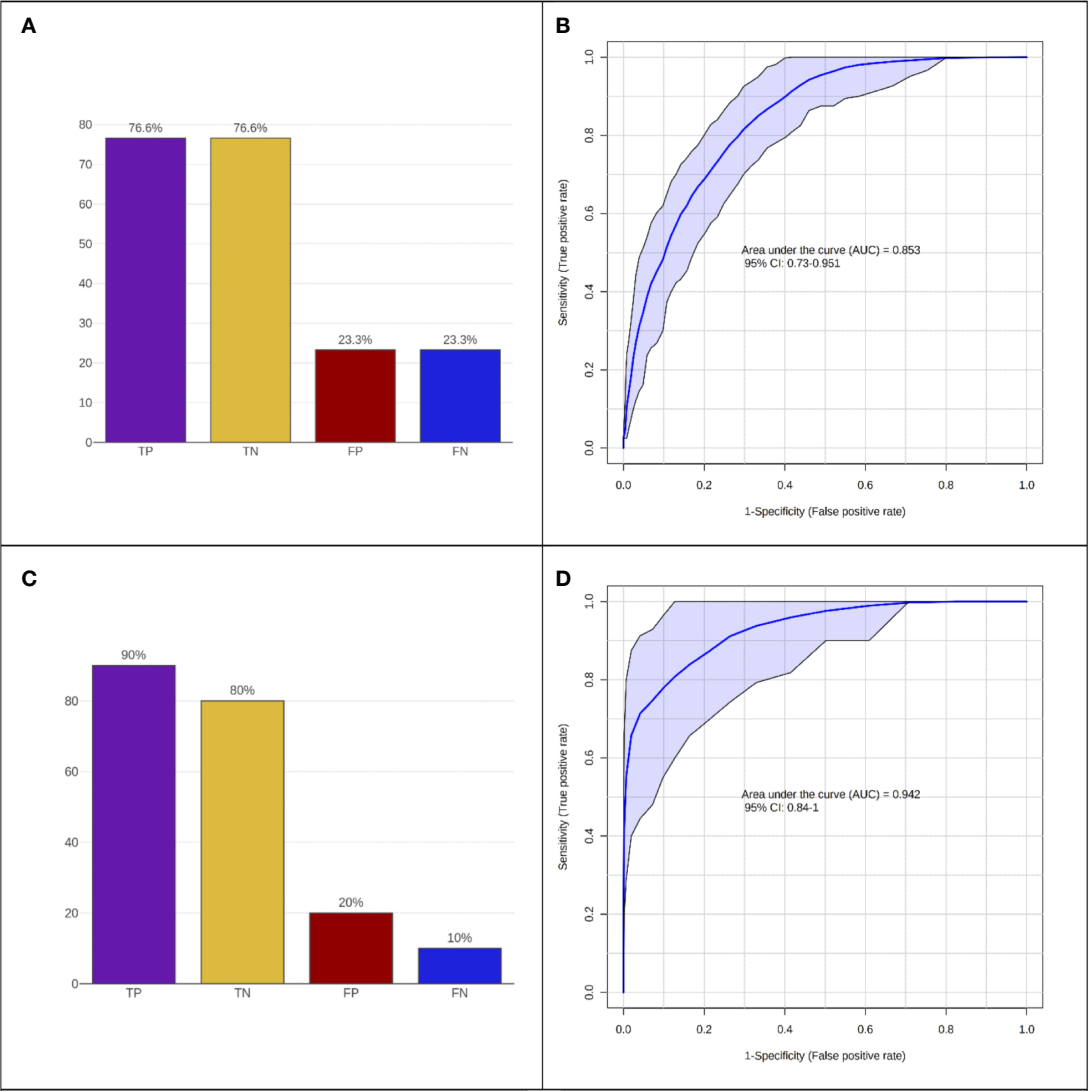
Performance of Random Forest classification models. **(A, B)** Controls vs. multiple sclerosis (MS) and **(C, D)** remitting MS vs. relapsing MS. **(A, C)** Accuracy expressed as = True Positive (TP), True Negative (TN), False Positive (FP), False Negative (FN) predictions. **(B, D)** ROC curve analysis.

The two PLS-DA analyses carried out for models (1) (controls vs. multiple sclerosis patients) and (2) (remitting vs. relapsing multiple sclerosis patients) to assess the variation in demographic/clinical, laboratory, and MRI features between groups. The results produced the Average Importance rankings of the variables, which are displayed in (**Figures 2A, B**, respectively). The top-5 ranking variables were employed to construct the Random Forest classifiers. Specifically, for model (1), they were: EBV-EBNA-1 IgG, BMI, PC, aC4 and TF; and for model (2), they were: TF, Angiopoietin-I, MCHC, Vitamin A and CD3+ T-cells. The performance of the classifiers was evaluated in terms of sensitivity and specificity, as indicated by the bar plots (**Figures 3A, C**, respectively), and by the ROC curves (**Figures 3B, D**, respectively). This resulted in an AUC of 0.853 (95% CI 0.730-0.951) for discriminating controls from multiple sclerosis patients and an AUC of 0.942 (95% CI 0.84-1.0) for the discrimination of remitting multiple sclerosis patients from relapsing ones. Both models, using a small number of variables, appeared to perform well in distinguishing the groups under exam with good accuracy.

## 4 Discussion

In the past years, accumulating evidence has shed light on the role of the coagulation system in the pathogenesis of multiple sclerosis (6, 16). In this study, we have undertaken a large-scale controlled analysis, comprehensive of demographic, clinical, laboratory (including coagulation/complement and vascular factors, viral/microbiological assay, fat-soluble vitamins and CBC with lymphocyte subsets) and radiological characteristics. Overall, we observed that blood and MRI alterations suggesting inflammatory-thrombotic processes occur in multiple sclerosis, especially during relapses, and can be influenced by demographic/clinical aspects and infectious status. We consequently built two predictive models to identify variables that played a possible role in distinguishing (1) controls vs. multiple sclerosis patients, and (2) remitting multiple sclerosis vs. relapsing multiple sclerosis patients.

Among the top-5 ranking variables (EBV-EBNA-1 IgG, BMI, PC, aC4 and TF) in the first model, EBV-EBNA-1 IgG was found to be the best one to discriminate controls from multiple sclerosis patients supporting the increasing body of evidence that suggests a preceding infection by EBV as a leading cause of multiple sclerosis (17, 18). Other serological markers (InfluenzaA IgG and Mycoplasma IgM) expressing previous or ongoing infection also differed between controls and multiple sclerosis patients, although they seemed to play a less relevant role. Furthermore, we found higher BMI in patients compared to controls, corroborating the growing evidence of a link between BMI and multiple sclerosis risk (19). Obesity is known to generate, through adipokines, a subtle pro-inflammatory systemic state that increases BBB permeability and triggers neuroinflammation (19). Indeed, we observed a positive association between BMI and C3, which in turn correlated with key coagulation factors such as fibrinogen and FX; this further supports the relationship between BMI and neuroinflammation/coagulation. We also found higher values of FVII, fibrinogen, D-dimer and FX in addition to one of the top-5 variables, i.e., PC which was higher in multiple sclerosis vs controls, thereby confirming recent findings on a dysregulation of PC pathway components in multiple sclerosis patients (20). In our cohort, PC values were associated with FVII and FX levels, which in turn correlated with PT, further supporting the role of the coagulation in multiple sclerosis pathophysiology.

Regarding the complement components, previous studies have reported their plasma/serum levels as either increased, reduced, or unchanged in multiple sclerosis patients with respect to controls (21, 22). We only found different levels of complement components for some of their activated forms (i.e., lower aC4 and higher aC9) in multiple sclerosis patients compared to controls but not between relapsing and remitting patients, consistent with more recent data reporting the absence of correlation between complement and disease activity in treatment-naïve early multiple sclerosis patients (22). Thus, our observations may contribute to the ongoing debate regarding the role of PC and complement in multiple sclerosis (20, 22).

Several differences emerged not only between patients and controls, but also between relapsing and remitting-multiple sclerosis patients. Our predictive model (2) identified the following variables as the top-5 ranking ones: TF, Angiopoietin-I, MCHC, Vitamin A and CD3+ T-cells. We observed lower levels of TF and Tie-2, as well as higher levels of Angiopoietin-I and VEGF in the relapsing-multiple sclerosis group. The Angiopoietin/Tie system controls endothelial cell survival and vascular maturation/stabilization, acting as a vascular specific ligand/receptor system (23). Altered Tie2 signaling has been linked to impairments in vascular function associated with many diseases, including cancer, cardiovascular diseases, and systemic inflammation (24). Moreover, in endotoxemic mice, reduced Tie2 signaling preceded signs of overt disseminated intravascular coagulation (25). Our findings suggest altered Angiopoietin-I/Tie2 signaling in the relapsing phase of multiple sclerosis patients which, to our knowledge, has not been yet investigated in multiple sclerosis and requires further in-depth studies. Similarly, the precise role in multiple sclerosis pathogenesis of fat-soluble vitamins such as Vitamin E, A and K - all considered as disease-modifying nutraceuticals - as well as their relationship with the gut microflora have not been thoroughly investigated (26). In our participants, the higher Vitamin A and K levels were found in relapsing- compared to either remitting patients or controls, and Vitamin K values negatively correlated with either CBV, CBF or MTT of active lesions while those of Vitamin A positively correlated with the number of relapses in previous 24- and 12 months. Moreover, within active gadolinium-enhancing lesions, the CBV positively correlated with PC while the MTT negatively correlated with TF. Furthermore, the CBV of NAWM and caudatum, as well as the CBF of putamen, were significantly lower in relapsing- compared to remitting-multiple sclerosis patients, suggesting that the global hypoperfusion may correlate with the overall blood flow deceleration due to inflammatory-thrombotic processes. The observation of deep grey matter hypoperfusion in patients with multiple sclerosis confirms the findings of previous studies (27–33). Some hypotheses have been proposed (27); hypoperfusion could be due to neuroaxonal loss as a consequence of demyelination processes, although in the majority of the studies no association between perfusion and brain atrophy was found (34–37). Other possible explanations (27) could include a reduction in energy demand or metabolic consumption (38, 39), primary ischemia (40), and cerebrovascular reactivity (41–43), and mitochondria alterations (44–46). Hypoperfusion could be preliminary to the development of neurodegenerative processes leading to brain atrophy (27).

It is noteworthy to point out that, for both models, the TF is in the top-5 variables that have a major role in this successful discrimination. In our cohort, systemic levels of TF were lower in relapsing compared to remitting patients and in multiple sclerosis vs controls, likely indicating its free, unbound in complex, form. TF is the key trigger of the coagulation cascade and formation of the TF : FVIIa complex activates both FX and FIX, with consecutive thrombin generation, fibrin deposition and platelet activation (47). Within the CNS, TF is predominantly expressed by astrocytes and mediates intracellular signaling via activation of protease-activated receptors (PARs) expressed in several cell types. BBB disruption, occurring in multiple sclerosis, exposes both perivascular and astrocyte TF to circulating clotting factors, which trigger the coagulation cascade. Furthermore, hemostasis components like fibrinogen, which leak into the brain parenchyma due to increased BBB permeability, are incorporated by astrocytes, leading to the formation of extensive fibrin(ogen) deposition. Finally, fibrinogen seems to activate microglia, leading to subsequent chemokines and cytokines production, macrophage and lymphocyte T recruitment, autoimmunity and reactive oxygen species generation. Impaired fibrinolysis increases fibrin deposition. All these processes lead to axonal damage, demyelination, and inhibition of remyelination (48). PARs, also widely expressed by either immune or nonprofessional immune cells such as platelets, link the coagulation system with the inflammatory response, controlling the immune response to viral infection (49). In particular, PAR-1 seems to induce pro-inflammatory and anti-inflammatory signaling under activation by thrombin or the anticoagulant activated PC (aPC), respectively (50, 51). However, the presence of TF and PC inhibitors in multiple sclerosis lesions suggests pro-inflammatory thrombin formation and suppression of the anti-inflammatory aPC pathways (48). Given the above, it has been hypothesized that coagulation activation at the level of BBB and neurovascular unit might induce and sustain inflammatory processes underlying the pathophysiology of multiple sclerosis. Indeed, TF expression has been found to increase, along with extensive fibrin deposition, in multiple sclerosis lesions (52). The findings in experimental autoimmune encephalomyelitis model also support the importance of vasculature damage and coagulation and their close relationship with neuroinflammation, neuroimmunology, and neurodegeneration (53–55).

Our observations on lower levels of TF in relapsing than in remitting multiple sclerosis patients might also relate to negative feedback due to higher levels of TF pathway inhibitor (TFPI), which have been found in progressive phenotypes of multiple sclerosis compared with relapsing-remitting multiple sclerosis (56). TFPI can interact with the transient complex TF/FVIIa/FXa or directly with the free FXa and is released from endothelial cells (with two forms, cell membrane-bound [TFPI-beta] and soluble in plasma) and platelets (TFPI-alfa). The predominant form TFPI-beta regulates the TF-mediated inflammatory responses through PARs signaling and can suppress the production of TNF-alfa and IL-6 and promote the increase of anti-inflammatory IL-10 (48, 57). Therefore, TFPI might exert a potential anti-inflammatory and neuroprotective role in the active forms of MS. Unfortunately, in this study, we did not measure the peripheral levels of TFPI and therefore we could not verify this hypothesis.

It should be taken into account that the coagulation activation has also the role of limiting pathogen spreading but some viruses, such as Herpes-virus, use this system to coat virally infected cells with fibrin reducing their recognition and killing by NK cells and phagocytes (58–60). In fact, TF expression is increased in HSV-infected endothelial cells of mice that produce higher viral amount compared to TF-negative infected cells (58).

Finally, in our study, CD3+ T-cell count is one of the top-5 variables in the model distinguishing relapsing vs. remitting- multiple sclerosis, and a lower lymphocyte count was found in multiple sclerosis compared to controls, albeit these parameters did not correlate with some coagulative/vascular or viral/microbiological markers. However, the relationship between the cytopenia, coagulopathy and viral infection has been recently confirmed in severe COVID-19 infection with elevated D-dimer (expressing the increased risk of thrombosis) together with lymphopenia and neutrophilia (61). Indeed, viral infections can result in a systemic lymphopenia, even though the underlying mechanisms are not completely understood (62).

The strengths of this study include the extensive number of laboratory and advanced radiological parameters using 3-T MRI evaluated in relapsing-remitting multiple sclerosis patients in the two different phases of the disease and the controlled design. This study has limitations. First, this was a non-longitudinal study which did not allow us to assess the dynamics of the relationship between the coagulation/complement biomarkers and the perfusion metrics and their interaction with the lesion activity over time. Second, the number of subjects included in this study was not very high, however, this allowed us to evaluate a relevant number of laboratory and MRI parameters. Third, since only relapsing-remitting multiple sclerosis patients were included in the analysis, study results cannot be generalized to the entire multiple sclerosis patient population. Fourth, there could be residual confounding of variables which we did not collect in our study and we could not demonstrate a causal relationship between the levels of TF and the other variables and the diagnosis of MS vs. controls or of relapsing vs. remitting MS. However, the prespecified exclusion criteria of the study and the relevant number of variables considered in the analyses could have reduced the risk of residual confounding. Furthermore, the two identified models appeared to significantly perform well in distinguishing the groups under exam (multiple sclerosis patients from controls and relapsing from remitting multiple sclerosis) with good accuracy. Given the exploratory nature of our analysis, there is the need of larger prospective studies to confirm our results and to obtain more reliable conclusions on the role of the identified variables, particularly of TF, in multiple sclerosis.

In conclusion, based on our two predictive models and the already recognized aspects of multiple sclerosis pathogenesis, we may assume that previous EBV infection, along with other conditions (obesity, altered gut microbiota, etc.), induces prolonged immune system dysfunction predisposing to the multiple sclerosis pathology. In this dis-immune state, multiple sclerosis exacerbations, accompanied by altered cerebral perfusion and inflammatory-thrombotic processes at both systemic and CNS level, could be determined by neurotropic infections together with consensual reactive immune responses. Circulating TF levels appear a promising pro-coagulative/vascular biomarker and a possible therapeutic target in relapsing-remitting multiple sclerosis.

## Data availability statement

The original contributions presented in the study are included in the article/**Supplementary Material**. Further inquiries can be directed to the corresponding author.

## Ethics statement

The study, including the informed consent form, was approved by the Ethics Committees of the IRCCS Regina Elena National Cancer Institute and of the Sapienza University of Rome. The patients/participants provided their written informed consent to participate in this study.

## Author contributions

TK: conceptualization, study design, funding acquisition, data interpretation, investigation, methodology, project administration, supervision, validation, writing-review/editing of the manuscript including figures and tables. SL: data interpretation, methodology, writing-review/editing of the manuscript including figures and tables. MC: writing-original draft, methodology, investigation, tables. MT: statistical analysis of the data, data interpretation, data curation, validation, tables, writing-review/editing of the manuscript. MF: data collection, dataset check, data interpretation. CL: data collection, dataset check, data interpretation. GD’A: data collection, dataset check, data interpretation. LC: conceptualization, study design, data interpretation, investigation. AS: data collection, dataset check, data interpretation. SZ: data collection, dataset check, data interpretation. MMF: data collection, dataset check, data interpretation. AC: data collection, dataset check, data interpretation. CP: data collection, dataset check, data interpretation. MM: data collection, dataset check, data interpretation. AM: data collection, dataset check, data interpretation. EG: data collection, dataset check, data interpretation. MS: data interpretation, investigation, methodology, supervision. MI: conceptualization, investigation, supervision, validation. All authors contributed to the article and approved the submitted version

## Supplementary material

Supplementary Material 2 (the second includes the study protocol and statistical analysis plan)
