# Supplementary material for: Tissue factor as a potential coagulative/vascular marker in relapsing-remitting multiple sclerosis
Source: Front Immunol. 2023 Jul 31;14:1226616. doi: 10.3389/fimmu.2023.1226616 (PMC10424925; doi:10.3389/fimmu.2023.1226616)
Supplement: Supplementary Material — 2 (the second includes the study protocol and statistical analysis plan) [file DataSheet_1.pdf]

Supplementary material

Tissue factor as a potential coagulative/vascular marker in relapsing-remitting multiple sclerosis

Tatiana Koudriavtseva, MD, PhD;[1,2] Svetlana Lorenzano, MD, PhD, MSc;[3] Maria Cellerino, MD, PhD;[2,4] Mauro Truglio, MSc;[5] Marco Fiorelli, MD, PhD;[3] Caterina Lapucci, MD, PhD[4;] Giovanna D'Agosto, MSc;[6] Laura Conti, MD;[5] Annunziata Stefanile, MSc;[5] Silvana Zannino, MD;[2] Maria Maddalena Filippi, MD;[7] Antonio Cortese, MD, PhD;[3] Carlo Piantadosi MD;[8] Marta Maschio, MD;[2] Andrea Maialetti, MSc;[2] Edvina Galiè, MD, PhD;[2] Marco Salvetti, MD, PhD;[9,10] and Matilde Inglese, MD. PhD.[11,4]

1 Medical Direction, IRCCS (Istituto di Ricovero e Cura a Carattere Scientifico) Regina Elena National Cancer Institute, Rome, Italy, tatiana.koudriavtseva@ifo.it;

2 Department of Clinical Experimental Oncology, IRCCS (Istituto di Ricovero e Cura a Carattere Scientifico) Regina Elena National Cancer Institute, IFO (Istituti Fisioterapici Ospitalieri), Rome, Italy, mariacellerino@hotmail.com, marta.maschio@ifo.it, andrea.maialetti@ifo.it, edvina.galie@ifo.it

3 Department of Human Neurosciences, Sapienza University of Rome, Italy, svetlana.lorenzano@gmail.com, marco.fiorelli@uniroma1.it

4 Department of Neuroscience, Rehabilitation, Ophthalmology, Genetics, Maternal and Child Health (DINOGMI, Dipartimento di Neuroscienze, Riabilitazione, Oftalmologia, Genetica e Scienze Materno-Infantili), University of Genoa, Genoa, Italy, lapuccicate@gmail.com, m.inglese@unige.it

5 Clinical Pathology and Cancer Biobank, IRCCS (Istituto di Ricovero e Cura a Carattere Scientifico) Regina Elena National Cancer Institute, Rome, Italy, mauro.truglio@ifo.it, laura.conti@ifo.it, stefanile.nunzia@gmail.com

6 Clinical Pathology and Microbiology, IRCCS (Istituto di Ricovero e Cura a Carattere Scientifico) San Gallicano Dermatological Institute, Rome, Italy, giovanna.dagosto@ifo.it

7 Neuroscience and Imaging, Fatebenefratelli Hospital, Isola Tiberina, Rome, Italy, filippi.mariam@gmail.com

8 Unità Operativa Complessa (UOC) Neurology, San Giovanni-Addolorata Hospital, Rome, Italy, cfpiantadosi@gmail.com.

9 Department Of Neuroscience Mental Health and Sensory Organs (NEMOS), Sapienza University, Rome, Italy, marco.salvetti@uniroma1.it

10 IRCCS Istituto Neurologico Mediterraneo Neuromed, Pozzilli, Italy

11 Department of Neurology, Mount Sinai Hospital, New York, USA, matilde.inglese@mssm.edu

Correspondence to: Svetlana Lorenzano, MD, PhD, MSc, FESO
Department of Human Neurosciences
Sapienza University of Rome,
Viale dell'Università 30, 00185
Rome, Italy
Telephone: 0039 349 2180282

Email: svetlana.lorenzano@gmail.com

Supplementary Tables: 6

Supplementary Methods

Laboratory data

A list of the laboratory biomarkers is reported in Supplementary Table 1.

Evaluation of ***complement and coagulation markers*** included serum/plasma concertation of D-Dimer, Fibrinogen, Factor II (FII), Factor VII (FVII), Factor VIII (FVIII), Factor X (FX), Prothrombin Time (PT), Activated Partial Thromboplastin Time (APTT), Antithrombin III (ATIII), Protein C (PC), Protein S (PS), von Willebrand Factor (VWF), C3, C4, Human Activated C4 (aC4), Activated Human C9 (aC9), Angiopoietin I, Angiopoietin II, Human Coagulation Factor III/Tissue Factor (TF), Vascular Endothelial Growth Factor (VEGF), Soluble Endothelial protein C receptor (sEPCR), Human Tie-2, P-Selectin/CD62P, E-selectin/CD62E, Endothelin-1, PLA2G7/PAF, Thrombomodulin/BDCA-3, fragment 1+2 of prothrombin (PF1/2) and of the thrombin-antithrombin III complex (TAT), Vitamin A, K, E and D and antiphospholipid antibodies (APLs)LP, including Anti-Cardiolipin (IgG, IgM), Anti-b2 Glycoprotein I (IgG, IgM), Anti-Prothrombin (IgG, IgM), and Anti-AnnexinV (IgG/IgM).

As per ***viral/microbiological serological assay***, we evaluated the concentration of Epstein-Barr (EBV) IgA/IgG/IgM, EBV-EBNA-1 IgG/IgM, EBV-VCA IgG/IgM, Chlamydia IgA/IgG/IgM, InfluenzaA IgA/IgG/IgM, InfluenzaB IgA/IgG/IgM, Herpes Simplex (HSV) Type1-2 IgG/IgM, HerpesSimplexType1 HSVType1 IgG/IgM, HerpesSimplexType2 HSVType2 IgG/IgM, Cytomegalovirus (CMV) IgG/IgM, Mycoplasma IgA/IgG/IgM, Varicella HerpesZoster (EZV) IgA/IgG/IgM, Ascaris IgG, Fascicola IgG, Schistosoma IgG.

For the ***blood cell count*** test, we analyzed the number of red blood cells (erythrocytes or RBC), white blood cells (leukocytes or WBC) including neutrophils (Neu) and lymphocytes (Lym), platelets (PLT), and the calculation of the neutrophil to lymphocyte ratio (NLR) and neutrophil to platelets ratio (PLR), hematocrit (HCT), hemoglobin (Hb), levels of the leukocyte formula (percentage of different white blood cells including neutrophils and lymphocytes), as well as the physical characteristics of the red blood cells by means of the following indices: MCV (mean corpuscular volume (MCV), MCH (mean corpuscular hemoglobin), MCHC (Mean Corpuscular Hemoglobin Concentration).

In the patient group alone, lymphocyte sub-populations count was measured including CD3+, CD8+, and CD4+ T-cells and the ratio between CD4 and CD8, CD19+ B-cells, and CD16/CD56 NK cells.

Supplementary Table 1. List of the laboratory biomarkers measured in the study

Abbreviation	Extended name	Unit
Coagulation and complement markers		
Fibrinogen	Fibrinogen	mg/dl
PT%	Prothrombin Time %	%
PT	Prothrombin Time	seconds
APTT	Activated Prothrombin Time	seconds
APTT-ratio	Activated Prothrombin Time - ratio	
PS	Protein S	%
PC	Protein C	%
FII	Factor II	%
FVII	Factor VII	%
FVIII	Factor VIII	%
FX	Factor X	%
vWF	von Willebrand Factor	%
AT III	Antithrombin	%
TAT	Thrombin – Antithrombin Complex	μg/l
DD	D-dimer	ng/ml
C3	Complement component 3	mg/dl
C4	Complement component 4	mg/dl
aC4	Activated C4	ng/ml
aC9	Activated C9	μg/ml
AngI	Angiopoietin I	pg/ml
AngII	Angiopoietin II	pg/ml
TF	Coagulation Factor III/Tissue Factor	pg/ml
VEGF	Vascular Endothelial Growth Factor	UM
Tie-2	Tie-2	UM
sEPCR	Soluble Endothelial protein C receptor	pg/ml
P-Sel	P-Selectin/CD62P	UM
E-Sel	E-selectin/CD62E	UM
End-1	Endothelin-1	UM
PLA2G7/PAF	PLA2G7/PAF	UM
Thr	Thrombomodulin/BDCA-3	UM
PF1/2	Prothrombin Fragment 1/2	pmol/L
VitA	Vitamin A	UM
VitK	Vitamin K	UM
VitE	Vitamin E	UM
VitD	Vitamin D	UM
ENA	Extractable Nuclear Antigen index	
Anti-card IgG	Anti-cardiolipin IgG	MPL
Anti-card IgM	Anti-cardiolipin IgM	MPL
Anti-β2 GPI IgG	Anti-Beta2 Glicoprotein I IgG	U/ml
Anti-β2 GPI IgM	Anti-Beta2Glicoprotein I IgM	U/ml
Anti-ptb IgG	Anti-prothrombin IgG	U/ml
Anti-ptb IgM	Anti-prothrombin IgM	U/ml
Anti-ann IgG	Anti-annexin IgG	U/ml
Anti-ann IgM	Anti-annexin IgM	U/ml

Viral/microbiological serological assays		
EB IgA	Epstein-Barr IgA	U/ml
EB IgG	Epstein-Barr IgG	U/ml
EB IgM	Epstein-Barr IgM	U/ml
EBV-EBNA IgG	Epstein Barr Virus-Epstein Barr Nuclear Antigen-1 IgG	U/ml
EBV-EBNA IgM	Epstein Barr Virus-Epstein Barr Nuclear Antigen -1 IgM	U/ml
EBV-VCA IgG	Epstein Barr Virus-Viral Capsid Antigen IgG	U/ml
EBV-VCA IgM	Epstein Barr Virus-Viral Capsid Antigen IgM	U/ml
Chl IgA	Chlamydia IgA	DU
Chl IgG	Chlamydia IgG	DU
Chl IgM	Chlamydia IgM	DU
InfA IgA	InfluenzaA IgA	DU
InfA IgG	InfluenzaA IgG	DU
InfA IgM	InfluenzaA IgM	DU
InfB IgA	InfluenzaB IgA	DU
InfB IgG	InfluenzaB IgG	DU
InfB IgM	InfluenzaB IgM	DU
HS 1-2 IgG	HerpesSimplexType1-2 IgG	DU
HS 1-2 IgM	HerpesSimplexType1-2 IgM	DU
HS 1 IgG	HerpesSimplexType1 IgG	DU
HS 1 IgM	HerpesSimplexType1 IgM	DU
HS 2 IgG	HerpesSimplexType2 IgG	DU
HS 2 IgM	HerpesSimplexType2 IgM	DU
Cyt IgG	Cytomegalovirus IgG	DU
Cyt IgM	Cytomegalovirus IgM	DU
Myc IgA	Mycoplasma IgA	DU
Myc IgG	Mycoplasma IgG	DU
Myc IgM	Mycoplasma IgM	DU
Var IgA	Varicella IgA	DU
Var IgG	Varicella IgG	DU
Var IgM	Varicella IgM	DU
Asc IgG	Ascaris IgG	DU
Fas IgG	Fascicola IgG	DU
Sch IgG	Schistosoma IgG	DU
Blood cell count		
WBC	White blood cells	ccx10^9/L
RBC	Red blood cells	ccx10^9/L
PLT	Platelets	ccx10^9/L
Hb	Hemoglobin	g/L
Htc	Hematocrit	%
MCV	Mean Corpuscular Volume	fL
MCH	Mean Corpuscolar Hemoglobin	pg
MCHC	Mean Corpuscular Hemoglobin Concentration	g/L
RDW	Red blood cells Distribution Width	%
MPV	Mean Platelet Volume	fL
PDW	Platelet Distribution Width	
Neu	Neutrophil	ccx10^9/L
Lym	Lymphocyte	ccx10^9/L
NLR	Neutrophil to lymphocyte ratio	
PLR	Platelets to lymphocyte ratio	
CD3+T	Cluster of Differentiation 3+ T cells	cells/ml
CD4+T	Cluster of Differentiation 4+ T cells	cells/ml
CD8+T	Cluster of Differentiation 8+ T cells	cells/ml
CD4/CD8	Cluster of Differentiation 4/Cluster of Differentiation 8	cells/ml
CD19+B	Cluster of Differentiation 19+ B cells	cells/ml

CD16/CD56	Cluster of Differentiation 16\Cluster of Differentiation 56 Natural Killer	cells/ml

Neuroimaging data

A list of the neuroimaging metrics is reported in Supplementary Table 2.

All MS patients underwent 3.0-T MRI within two weeks of enrollment.

<u>Lesion analysis</u>

White matter lesions (WML) were manually segmented by using Jim software (Xinapse, Version 7.0) on 3D FLAIR, 3D T1 MPRAGE, T1SE 3 mm-resliced images. Brain volumes segmentation was performed on 3D T1 MPRAGE images after nu-correction and lesion filling by using Matlab-based Statistical Parametric Mapping Toolbox (SPM CAT12; http://www.fil.ion.ucl.ac.uk/spm).

<u>Perfusion analysis</u>

We used dynamic-susceptibility contrast-enhanced (DSC) perfusion technique acquired during the first pass of gadolinium to estimate perfusion features inside the damaged tissue of relapsing- and remitting-MS patients. DSC MR images were acquired on the axial plane during the first pass of a standard-dose bolus (0.1 mmol/kg) of gadoterate meglumine (Dotarem; Guerbet Laboratories, Villepinte, France) with a gradient-echo T2-weighted echoplanar imaging sequence. The contrast agent was injected at a rate of 3,5 mL/sec, followed by a 20-mL bolus of saline also at a rate of 3,5 mL/sec. A total of 60 images were acquired at 1-sec intervals, with the injection occurring at the fifth image, for a total acquisition time of 2 min 16 s. The imaging parameters were as follows: TR/TE = 2140/30 ms, flip angle = 30°, slice thickness = 4 mm, FOV = 280 mm, matrix = 128x128. Perfusion maps (CBF, CBV, MTT) were created by using nordiICE software package. Deep gray matter (DGM) structures segmentation was obtained by using FIRST software. After co-registration of structural images with perfusion maps, CBV, CBF and MTT metrics were extracted from enhancing and non-enhancing WML, DMG and NAWM**.**

A leakage correction analysis was also performed, in order to correct for contrast agent extravasation. Due to partial volume effects in the measured Arterial Input Function (AIF) combined with non-linear dose-response conditions in DSC PWI, the obtained CBF and CBV values do not generally reflect absolute perfusion and blood volume values, and were consequently reported in arbitrary units.

Supplementary Table 2. List of the radiological (MRI) metrics measured in the study

**Metric**	**Explored areas**	**Unit**
Lesion analysis		

Lesion volumes	FLAIR lesions<br>T1 lesions<br>Active (Gd+) lesions	ml
Number of lesions		-
Perfusion metric analysis		
MTT	FLAIR lesions<br>Active (Gd+) lesions<br>NAWM<br>Caudate *(right, left, global)*<br>Thalamus *(right, left, global)*<br>Globus pallidus *(right, left, global)*<br>Putamen *(right, left, global)*	n.a.[#]
MTT leakage°		
CBV putamen		
CBV leakage°		
CBF		
CBF leakage°		

CBF=cerebral blood flow; CBV= cerebral blood volume; FLAIR=fluid-attenuated inversion recovery; Gd+=gadolinium enhancing; MTT=mean transit time; NAWM=normal appearing white matter; n.a. not available

° A leakage correction analysis was also performed, in order to correct for contrast agent extravasation.

Due to partial volume effects in the measured Arterial Input Function (AIF) combined with non-linear dose-response conditions in DSC PWI, the obtained CBF and CBV values do not generally reflect absolute perfusion and blood volume values, and were consequently reported in arbitrary units.

Supplementary Results

All between-groups (both statistically significant and non-significant) differences in serum/plasma laboratory variables are reported in Supplementary Table 3.

Supplementary Table 3. Between group differences in terms of laboratory biomarkers in controls and remitting- and relapsing-multiple sclerosis patients

A - Coagulation/complementbio markers	Controls (n=30)		Remitting-MS (n=30)		Relapsing-MS (n=30)		
	Mean (SD)	Median (IQR)	Mean (SD)	Median (IQR)	Mean (SD)	Median (IQR)	*P-value*[*]
PT	1.032 (0.095)	1.01 (0.105)	1.031 (0.086)	1.03 (0.078)	1.034 (0.067)	1.025 (0.1)	0.98
PT%	96.867 (12.292)	99 (15.5)	97 (11.671)	96 (12)	95.867 (9.104)	96.5 (13.75)	0.91
APTT	31.017 (2.451)	30.5 (3.45)	30.023 (2.873)	30.25 (2.95)	31.023 (2.499)	30.85 (3.625)	0.936
APTT-ratio	0.979 (0.075)	0.965 (0.095)	0.955 (0.077)	0.95 (0.1)	0.972 (0.083)	0.975 (0.115)	0.486
PS	91.567 (14.5)	88.5 (24.25)	80.367 (22.338)	81 (30)	87.267 (16.432)	91.5 (17.25)	0.058
PC	99.13 (14.44)	97.5 (25.25)	110.66 (18.07)	116.5 (28.5)	116.86 (20.74)	117 (30)	**0.001**[a]
FII	102.111 (10.5)	102 (9.5)	104.833 (12.647)	105 (13.5)	98.767 (13.505)	97 (14.25)	0.169
FVII	82.264 (26.221)	85.55 (33)	90.041 (25.071)	88.8 (28.95)	89.679 (22.27)	89.9 (24.9)	0.469
FVIII	118.103 (23.966)	116 (33)	125.433 (28.441)	129 (30)	122.933 (32.601)	119 (33)	0.609
FX	88.397 (12.614)	87.4 (15.175)	95.307 (15.972)	93.55 (21.525)	94.417 (13.963)	93.55 (14.225)	0.13
VWF	123.28 (48.699)	108.2 (53.85)	140.303 (55.532)	137.85 (53.525)	121.914 (43.678)	118.7 (36.675)	0.251
AT	105.6 (7.295)	106 (9.25)	107.2 (9.045)	107 (10.25)	106.967 (11.149)	107.5 (12.75)	0.772
TAT	1.77 (1.856)	1.25 (1.275)	2.735 (4.554)	1.5 (1.55)	1.934 (1.277)	1.6 (1.7)	0.503

DD	205.9 (89.215)	192.5 (73.75)	293.367 (198.629)	250 (133.5)	286.9 (150.555)	233.5 (193.75)	0.073
C3	109.067 (15.37)	109 (18)	119.47 (24.6)	115.5 (32)	114.33 (18.9)	114 (24.25)	0.286
C4	25.23 (18.1)	22.5 (7.75)	23.6 (6.62)	23 (6)	23.07 (5.15)	24 (5.75)	0.703
aC4	5.78 (5.21)	4.75 (1.725)	2.75 (1.43)	2.3 (1.125)	3.91 (3.13)	2.85 (2.107)	**0.001**[b]
aC9	3.38 (1.13)	3.4 (1.675)	4.91 (3.03)	4.3 (3.3)	5.42 (4.13)	4.4 (3.54)	**0.019**[c]
AngI	9.40 (4.04)	8.38 (4.34)	7.31 (2.96)	7.26 (2.84)	10.57 (4.83)	9.76 (5.75)	**0.009**[d]
AngII	2943.04 (981.37)	2600 (1095)	2631.82 (1007)	2360 (1202.5)	3258.27 (1358.26)	2953.5 (1385.5)	0.11
TF	110.85 (56.31)	120.69 (101.11)	94.06 (55.83)	99.23 (104.22)	45.03 (37.66)	22.72 (58.67)	**<0.001**[e]
VEGF	32.07 (23.83)	24.5 (24)	26.03 (12.89)	23 (15.75)	41.82 (31.98)	33 (39)	0.224
Tie-2	49.60 (17.58)	43.15 (10.775)	46.45 (17.36)	40.85 (22.425)	34.42 (19.94)	35.7 (25.3)	**0.002**[f]
sEPCR	21.97 (21.82)	13.1 (29.5)	25.85 (24.85)	17 (22.95)	22.22 (19.44)	15.35 (24.7)	0.860
P-Sel	245.85 (120.48)	197 (156)	216.26 (82.15)	216.5 (131)	169.41 (173.51)	160 (215.4)	**0.020**[g]
E-Sel	170.99 (57.49)	153.995 (90)	159.02 (84.42)	149.6 (156.145)	127.21 (100.84)	127.05 (175.475)	0.122
End-1	24.00 (8.44)	23 (8)	24.17 (8.20)	26 (12)	23.68 (19.99)	24 (28.825)	0.965
PLA2G7/PAF	138.71 (39.93)	139.7 (62.7)	144.51 (50.41)	149.1 (62.5)	146.88 (48.38)	150.75 (63.15)	0.787
Thr	2060.24 (862.96)	1829 (810)	2377.11 (628.72)	2352.5 (1020.2)	2678.30 (1610.63)	2089.5 (1206)	0.198
PF 1/2	158.433 (60.584)	158.5 (84.5)	190.433 (81.946)	173.5 (122.5)	170.067 (69.169)	170.5 (94.25)	0.217
VitA°	40.75 (18.15)	32.68 (21.925)	43.15 (12.77)	41.63 (12.43)	105.18 (50.07)	100.8 (101.13)	**<0.001**[h]
VitK°	163.89 (110.80)	126.49 (119.56)	166.75 (107.20)	148.405 (167.115)	295.51 (64.84)	272.455 (299.222)	**<0.001**[i]
VitE°	812.85 (124.21)	788.02 (105.73)	787.11 (120.43)	791.975 (145.188)	849.13 (188.01)	742.3 (840.653)	0.590
VitD°	19.43 (7.95)	21 (12.2)	24.60 87.66)	24.25 (10.05)	22.45 (6.12)	22.3 (7.8)	0.470
ENA	-	-	0.23 (0.164)	0.2 (0)	0.325 (0.514)	0.2 (0)	0.5
Anti-card. IgG	2.17 (1.71)	1.6 (0)	2.43 (1.72)	1.6 (0.75)	2.37 (2.58)	1.6 (0.025)	0.146
Anti-card. IgM	2.26 (3.44)	0.75 (2.275)	3.41 (6.41)	0.65 (2.15)	2.91 (5.80)	0.6 (2.5)	0.790
Anti-β2 IgG	1.66 (0.83)	1.4 (0)	1.67 (0.79)	1.4 (0.15)	1.73 (1.10)	1.4 (0)	0.558
Anti-β2 IgM	2.21 (3.04)	0.9 (1.95)	2.82 (5.11)	0.9 (1.6)	2.28 (3.59)	0.65 (1.8)	0.812
Anti-ptb. IgG	5.89 (4.61)	4.4 (3.9)	5.14 (2.23)	4.8 (2.375)	4.68 (2.89)	4.05 (1.925)	0.336
Anti-ptb IgM	4.87 (6.63)	2.4 (1.875)	3.15 (2.96)	2 (2.05)	5.86 (12.52)	2.2 (1.875)	0.642
Anti-ann IgG	2.61 (1.78)	0.9 (0.875)	3.64 (4.97)	1.1 (0.6)	3.02 (2.93)	0.9 (0.55)	0.925
Anti-ann IgM	2.45 (6.83)	1.95 (1.725)	1.14 (0.68)	1.95 (1.275)	1.28 (1.39)	2.1 (1.575)	0.815

**B – Serological viral / microbiological assay**	**Controls (n=30)**		**Remitting-MS (n=30)**		**Relapsing-MS (n=30)**		
	**Mean (SD)**	**Median (IQR)**	**Mean (SD)**	**Median (IQR)**	**Mean (SD)**	**Median (IQR)**	***P-value****[*]**
EB IgA	1.47 (1.19)	1.24 (0.438)	1.61 (1.23)	1.24 (0.93)	1.55 (0.99)	1.195 (1.083)	0.800
EB IgG	12.65 (38.39)	1.65 (1.85)	10.63 (28.098)	2.3 (3.025)	2.86 (3.13)	1.7 (0.75)	0.117
EB IgM	6.62 (7.39)	4.35 (3.5)	3.87 (1.86)	3.45 (2.6)	4.30 (2.55)	3.75 (2.75)	0.191
EBV-EBNA IgG	26.81 (7.48)	27.65 (8.325)	31.05 (6.35)	32.15 (6.3)	31.89 (4.66)	32 (5.3)	**0.014**[l]
EBV-EBNA IgM	4.79 (4.37)	3.35 (2.55)	5.95 (4.79)	4.9 (6.075)	5.35 (4.37)	4.65 (6.575)	0.622

EBV-VCA IgG	47.14 (20.32)	50.2 (27.175)	56.01 (16.07)	57.9 (18.05)	57.17 (19.49)	66.3 (25.1)	0.070
EBV-VCA IgM	7.47 (6.91)	4.7 (4.375)	5.88 (2.51)	4.85 (3.85)	7.24 (11.86)	5.65 (3.275)	0.635
Chl IgA	2.60 (1.99)	1.85 (1.775)	4.14 (5.07)	2.25 (2.25)	2.50 (1.58)	2.2 (1.575)	0.570
Chl IgG	6.65 (6.01)	4.5 (10.875)	7.21 (7.54)	6 (8.925)	7.01 (5.57)	5.75 (10.025)	0.870
Chl IgM	4.28 (3.47)	3.15 (2.375)	3.62 (2.27)	2.9 (2.525)	3.07 (1.37)	2.6 (2.1)	0.580
InfA IgA	2.49 (3.23)	1.45 (0.825)	2.91 (2.72)	2.1 (1.725)	1.71 (1.23)	1.35 (0.75)	**0.029**[n]
InfA IgG	11.59 (7.61)	8.75 (9.275)	15.87 (7.49)	15.7 (12.375)	17.88 (11.98)	12.45 (21.45)	**0.041**[m]
InfA IgM	3.24 (4.64)	1.9 (1.425)	2.22 (2.99)	1.55 (1.55)	1.89 (2.67)	1.4 (1.375)	0.350
InfB IgA	1.81 (1.64)	1.15 (1.95)	1.90 (1.76)	1.9 (1.1)	1.68 (1.42)	1.2 (1.55)	0.282
InfB IgG	15.12 (7.55)	14.75 (5.05)	19.30 (9.68)	17.3 (12.075)	14.08 (8.99)	13.1 (9.75)	0.059
InfB IgM	1.99 (3.66)	0.8 (0.9)	1.18 (1.93)	0.7 (0.675)	1.18 (2.28)	0.65 (0.75)	0.601
HS 1-2 IgG	12.93 (12.23)	11.25 (20.25)	16.79 (12.78)	17 (22.25)	17.53 (12.79)	17.4 (23.7)	0.320
HS 1-2 IgM	5.85 (5.65)	4.1 (5.575)	3.78 (2.74)	2.95 (3.475)	4.61 (4.25)	2.85 (4.975)	0.480
HS 1 IgG	21.43 (18.84)	18.7 (34.875)	24.49 (19.83)	20.9 (41.125)	26.11 (19.18)	27.85 (39.775)	0.545
HS 1 IgM	4.65 (3.99)	2.95 (2.525)	3.84 (2.96)	2.8 (3.1)	4.01 (4.22)	2.3 (2.925)	0.201
HS 2 IgG	7.03 (10.36)	2.15 (4.1)	7.45 (11.38)	3.2 (3.85)	8.03 (9.42)	4.45 (7.4)	0.135
HS 2 IgM	4.90 (6.26)	2.8 (4.675)	2.96 (2.52)	1.95 (2.8)	3.97 (3.81)	2.75 (4.15)	0.651
Cyt IgG	32.81 (37.74)	21.65 (63.7)	37.51 (34.08)	45.4 (62.325)	29.06 (34.27)	9.35 (58.4)	0.419
Cyt IgM	23.34 (46.49)	5.6 (14.7)	12.84 (22.58)	4 (7.4)	18.62 (62.78)	2.9 (5.15)	0.431
Myc IgA	7.21 (5.12)	6.15 (3.65)	7.03 (2.65)	6.7 (4.025)	5.46 (2.49)	4.9 (1.675)	0.060
Myc IgG	13.38 (10.04)	10.95 (10.875)	13.36 (10.47)	12 (7.375)	14.07 (16.14)	10.3 (10.05)	0.862
Myc IgM	8.11 (7.02)	6.8 (4.6)	5.69 (2.57)	4.95 (3.925)	4.97 (1.88)	4.55 (2.125)	0.055
Var IgA	7.18 (6.37)	5.25 (4.575)	7.74 (7.72)	4.25 (6)	5.63 (4.35)	4.05 (6.4)	0.767
Var IgG	17.08 (4.36)	18.3 (4.525)	16.08 (6.19)	16.7 (6.3)	19.18 (2.85)	18.75 (2.625)	0.092
Var IgM	3.86 (3.79)	2.65 (2.65)	5.50 (7.60)	2.4 (3.125)	2.85 (1.60)	2.45 (1.475)	0.596
Asc IgG	7.02 (2.62)	7 (4.2)	7.22 (3.46)	6.1 (4.625)	5.76 (3.22)	5 (3.325)	0.055
Fas IgG	2.27 (1.63)	1.8 (0.525)	2.31 (0.99)	2.05 (1.075)	1.81 (0.79)	1.6 (1.05)	0.067
Sch IgG	4.79 (1.74)	4.6 (2.225)	4.75 (2.28)	4.15 (2.25)	3.86 (1.41)	3.4 (0.65)	**0.023**[o]

**C - Complete blood cell count**	**Controls (n=30)**		**Remitting-MS (n=30)**		**Relapsing-MS (n=30)**		
	**Mean (SD)**	**Median (IQR)**	**Mean (SD)**	**Median (IQR)**	**Mean (SD)**	**Median (IQR)**	***P-value***[*]
WBC	5.91 (1.53)	5.87 (1.862)	6.29 (1.68)	6.165 (2.35)	6.41 (2.40)	5.8 (2.383)	0.560
RBC	4.71 (0.38)	4.585 (0.567)	4.59 (0.47)	4.59 (0.68)	4.59 (0.36)	4.565 (0.498)	0.445
Plt	251.80 (63.79)	251 (65.5)	251.46 (68.81)	251 (85.25)	255.16 (56.33)	242 (76)	0.969
Hb	14.05 (1.13)	13.8 (1.625)	13.49 (1.21)	13.3 (1.45)	13.63 (1.23)	13.5 (1.85)	0.182
Htc	42.42 (3.08)	41.55 (3.55)	40.95 (3.47)	40.35 (3.625)	40.75 (3.34)	40.65 (4.4)	0.109
MCV	90.39 (6.40)	91.9 (4.275)	89.55 (6.16)	89.65 (7.575)	88.27 (4.98)	88.3 (7)	0.377
MCH	29.94 (2.47)	30.5 (1.875)	29.51 (2.34)	29.5 (2.625)	29.73 (1.88)	29.75 (2.825)	0.754
MCHC	33.11 (0.79)	33.2 (0.875)	32.94 (0.66)	32.85 (0.775)	33.45 (0.74)	33.2 (1.25)	**0.027**[p]
RDW	13.493 (0.751)	13.4 (0.775)	13.667 (1.004)	13.45 (0.8)	13.5 (0.859)	13.3 (0.75)	0.68

MPV	9.247 (1.098)	9.15 (1.6)	9.127 (1.084)	9.05 (1.3)	9.247 (1.21)	9.15 (1.825)	0.89
PDW	16.93 (0.594)	16.9 (0.6)	16.94 (0.505)	16.9 (0.75)	16.757 (0.309)	16.7 (0.375)	0.31
Neu	3.34 (1.16)	3.29 (1.222)	3.79 (1.40)	3.475 (0.927)	4.16 (2.14)	3.75 (1.748)	0.155
Lym	1.91 (0.55)	1.82 (0.607)	1.86 (0.57)	1.84 (0.8)	1.49 (0.60)	1.48 (0.968)	**0.014**[q]
Neu/Lym	1.85 (0.68)	1.795 (0.995)	2.24 (1.11)	2.15 (1.08)	3.42 (2.51)	2.515 (2.23)	**0.005**[r]
Plt/Lym	139.87 (43.82)	132.345 (63.752)	149.16 (66.38)	143.23 (80.91)	227.66 (2.2.92)	173.545 (108.295)	**0.016**[s]
CD3+T	-	-	1.43 (0.48)	1.406 (0.764)	1.11 (0.51)	1.024 (0.528)	**0.015**
CD4+T	-	-	0.92 (0.29)	0.892 (0.43475)	0.73 (0.38)	0.653 (0.322)	**0.043**
CD8+T	-	-	0.47 (0.25)	0.405 (0.291)	0.34 (0.18)	0.336 (0.257)	**0.028**
CD4/CD8	-	-	2.32 (0.95)	2.315 (1.31)	2.50 (1.42)	2.34 (1.5)	0.903
CD19+B	-	-	0.30 (0.15)	0.275 (0.17)	0.22 (0.13)	0.223 (0.128)	**0.025**
CD16/CD56	-	-	0.22 (0.11)	0.171 (0.146)	0.21 (0.87)	0.189 (0.135)	0.860

IQR=interquartile range; MS=multiple sclerosis; SD=standard deviation; for the abbreviations and units of all laboratory parameters, see Supplementary Table 1.
° In the context of vitamin assessment, data were available in 57 subjects for Vit A, K, E (22 contrls, 18 remitting- and 17 relapsing- MS patients) and in 37 subjects for Vit D (11 controls, 4 remitting MS, 22 relapsing MS patients).
[*] Kruskall-Wallis or ANOVA according to the variable distribution
Significant differences are reported in bold; statistically significant post-hoc analysis details are described below:
[a] *P*=0.042 for controls vs Remitting-MS; p=0.001 for controls vs Relapsing-MS
[b] *P*=0.002 for controls vs Remitting-MS; p=0.008 for controls vs Relapsing-MS; p=0.039 for Relapsing-MS vs Remitting-MS
[c] *P*=0.022 for controls vs Remitting-MS; p=0.017 for controls vs Relapsing-MS
[d] *P*=0.005 for Relapsing-MS vs Remitting-MS
[e] *P*<0.001 for Relapsing-MS vs controls; p<0.001 for Relapsing-MS vs Remitting-MS
[f] *P*<0.001 for Relapsing-MS vs controls; p=0.032 for Relapsing-MS vs Remitting-MS
[g] *P*=0.013 for Relapsing-MS vs controls; p=0.032 for Relapsing-MS vs Remitting-MS
[h] *P*<0.001 for Relapsing-MS vs controls; p<0.001 for Relapsing-MS vs Remitting-MS
[i] *P*<0.001 for Relapsing-MS vs controls; p<0.001 for Relapsing-MS vs Remitting-MS
[l] *P*=0.017 for controls vs Remitting-MS; p=0.011 for controls vs Relapsing-MS
[m] *P*=0.037 for controls vs Remitting-MS; p=0.037 for controls vs Relapsing-MS
[n] *P*=0.014 for Relapsing-MS vs Remitting-MS
[o] *P*=0.014 for Relapsing-MS vs controls; p=0.038for Relapsing-MS vs Remitting-MS
[p] *P*=0.023 for Relapsing-MS vs Remitting-MS
[q] *P*=0.020 for Relapsing-MS vs controls; p=0.047 for Relapsing-MS vs Remitting-MS
[r] *P*=0.002 for Relapsing-MS vs controls; p=0.049 for Relapsing-MS vs Remitting-MS
[s] *P*=0.025 for Relapsing-MS vs controls; p=0.026 for Relapsing-MS vs Remitting-MS

Subgroup analysis by sex on laboratory markers were also performed. Between-sex differences in laboratory biomarkers for the entire cohort and patients only are reported in Supplementary Table 4.

Supplementary Table 4. Between-sex differences in laboratory biomarkers in the entire cohort and in relapsing- and remitting-multiple sclerosis patients' groups only.

		**All subjects (N=90)**				**Patients only (N=60)**			
		**N**	**Mean (SD)**	**Median (IQR)**	**p-value**	**n**	**Mean (SD)**	**Median (IQR)**	***P*-value**
**RBC (ccx10^9/L)**	**F**	70	4.52 (0.35)	4.49 (0.413)	**< .001**	47	4.473 (0.336)	4.47 (0.475)	**<0.001**
	**M**	20	5.02 (0.36)	5.01 (0.462)		13	5.022 (0.409)	4.95 (0.56)	
**PLT (ccx10^9/L)**	**F**	70	261.21 (62.1)	249 (76.75)	**0.042**	47	263.9 (60.49)	249 (75)	**0.016**
	**M**	20	223.4 (55.89)	226 (94.25)		13	215.08 (55.52)	207 (90)	
**Hb (g/L)**	**F**	70	13.37 (1.043)	13.35 (1.15)	**< .001**	47	13.238 (1.091)	13.1 (1.25)	**<0.001**
	**M**	20	14.98 (0.86)	14.95 (1.25)		13	14.754 (0.867)	14.7 (0.8)	
**Htc (%)**	**F**	70	40.40 (2.9)	40.35 (2.87)	**< .001**	47	39.981 (3.06)	40 (3.85)	**<0.001**

	**M**	20	44.78 (2.61)	44.9 (4.47)		13	44.015 (2.561)	43.3 (2.9)	
**MCHC (g/L)**	**F**	70	33.08 (0.76)	33 (0.87)	**0.025**	47	33.104 (0.704)	32.9 (0.8)	0.063
	**M**	20	33.48 (0.687)	33.55 (1)		13	33.546 (0.816)	33.6 (1)	
**RDW (%)**	**F**	70	13.66 (0.90)	13.5 (0.87)	**0.027**	47	13.709 (0.946)	13.5 (1)	**0.024**
	**M**	20	13.17 (0.63)	13.1 (0.72)		13	13.131 (0.733)	13 (0.7)	
**PLR**	**F**	69	183.5 (143.6)	155.23 (82.39)	**0.015**	46	204.01 (169.9)	171.47 (71.8)	**0.019**
	**M**	20	134.67 (62.24)	109.49 (59.77)		13	136.26 (72.01)	109.13 (20.96)	
**PT%**	**F**	69	95.304 (11.26)	96 (13)	**0.029**	46	96.043 (10.82)	96.5 (12)	0.621
	**M**	20	100.95 (8.92)	101 (10)		13	97.769 (8.861)	96 (10)	
**PT (seconds)**	**F**	70	1.042 (0.086)	1.035 (0.1)	**0.024**	47	1.036 (0.08)	1.03 (0.09)	0.565
	**M**	20	0.999 (0.059)	0.99 (0.07)		13	1.019 (0.062)	1.03 (0.07)	
**DD (ng/ml)**	**F**	70	267.93 (132.8)	241 (127.5)	**0.033**	47	292.39 (143.7)	254 (143)	0.114
	**M**	20	241.5 (223.39)	178.5 (115)		13	282 (266.332)	183 (170)	
**PS (%)**	**F**	70	83.614 (17.12)	88 (21.25)	**0.006**	47	81.723 (18.75)	89 (26)	0.125
	**M**	20	96.15 (20.08)	103.5 (28.5)		13	91.385 (22.16)	96 (36)	
**FII (%)**	**F**	67	100.3 (12.34)	100 (13.5)	**0.006**	47	100.3 (12.96)	100 (13)	**0.038**
	**M**	20	107.25 (11.58)	106 (5.5)		13	107.23 (13.74)	106 (10)	
**FVII (%)**	**F**	62	83.085 (22.14)	82.5 (28.2)	**0.002**	44	85.29 (19.85)	83.9 (26.675)	**0.027**
	**M**	16	105.64 (24.78)	102.5 (21.55)		12	106.6 (28.609)	99.55 (34.275)	
**C3 (mg/dl)**	**F**	70	111.69 (20.83)	109.5 (25.25)	**0.007**	47	114.47 (22.86)	113 (30)	**0.045**
	**M**	20	123.4 (15.132)	122.5 (26.5)		13	125.69 (15.9)	126 (29)	
**Anti-ptb IgM (U/ml)**	**F**	68	4.713 (7.339)	2.3 (2.7)	**0.01**	45	4.249 (7.335)	2.3 (2.7)	**0.007**
	**M**	20	4.225 (10.909)	1.6 (1.075)		13	5.2 (13.59)	1.5 (0.8)	
**Anti-ann IgM (U/ml)**	**F**	68	1.876 (4.62)	1.1 (0.8)	**0.019**	45	1.344 (1.183)	1.1 (0.7)	**0.009**
	**M**	20	0.81 (0.445)	0.8 (0.7)		13	0.738 (0.336)	0.8 (0.4)	
**C4 (mg/dl)**	**F**	66	4.559 (4.204)	3.3 (2.85)	**0.011**	43	3.643 (2.625)	2.8 (1.15)	**<0.001**
	**M**	20	2.97 (1.722)	2.3 (1.85)		13	2.308 (1.621)	1.9 (0.6)	
**FX (%)**	**F**	70	90.85 (14.21)	88.9 (18.725)	**0.014**	47	92.906 (15.04)	92 (18.55)	**0.043**
	**M**	20	99.22 (13.56)	98.6 (14.05)		13	101.93 (12.36)	101 (17.7)	
**EB IgA (U/ml)**	**F**	68	1.71 (1.23)	1.28 (1.04)	**0.006**	47	1.735 (1.21)	1.29 (1.035)	**0.027**
	**M**	20	0.999 (0.39)	1.095 (0.358)		13	1.048 (0.305)	1.1 (0.22)	
**EB IgM (U/ml)**	**F**	68	5.281 (5.071)	4 (2.925)	**0.018**	47	4.128 (1.977)	3.7 (2.55)	0.247
	**M**	20	3.58 (2.57)	2.8 (1.975)		13	3.938 (3.059)	2.7 (2.3)	
**EBV-VCA IgM (U/ml)**	**F**	68	7.435 (8.895)	5.8 (3.35)	**0.021**	47	7.164 (9.472)	5.9 (3.1)	**0.026**
	**M**	20	4.87 (3.217)	3.8 (3.9)		13	4.385 (2.669)	3.8 (2.9)	
**HS 1 IgM (DU)**	**F**	68	4.525 (3.982)	2.85 (2.775)	**0.016**	47	4.251 (3.805)	2.7 (3)	**0.023**
	**M**	20	2.915 (2.424)	1.95 (1.675)		13	2.754 (2.666)	1.7 (0.7)	
**Cyt IgM (DU)**	**F**	68	21.8 (51.79)	5.45 (10.65)	**< .001**	47	17.913 (51.79)	4.6 (7.3)	**0.019**
	**M**	20	5.76 (16.851)	1.05 (1.75)		13	7.838 (20.797)	1 (2.4)	
**Myc IgM (DU)**	**F**	68	6.628 (4.911)	5.45 (3.825)	**0.016**	47	5.638 (2.379)	5.2 (3.3)	0.053
	**M**	20	4.805 (2.473)	3.85 (2.35)		13	4.215 (1.364)	3.7 (0.9)	
**Var IgG (DU)**	**F**	68	16.999 (5.082)	17.8 (4.25)	**0.04**	47	17.068 (5.345)	17.8 (3.9)	0.057
	**M**	20	19.03 (3.382)	19.65 (3.825)		13	19.685 (2.995)	20.4 (5.3)	
**Sch IgG (DU)**	**F**	68	4.644 (2.007)	3.85 (2.225)	0.091	47	4.523 (2.071)	3.6 (2)	**0.043**
	**M**	20	3.84 (1.23)	3.45 (1.4)		13	3.523 (1.102)	3.2 (1.1)	
	**M**	-	-	-		13	20.615 (5.44)	19 (4)	
	**M**	-	-	-		13	27.85 (17.53)	22 (8)	

Anti-ann=anti-annexin; Anti-ptb IgM=anti-prothrombin; C3=complement component 3; C4=complement component 4; Cyt=Cytomegalovirus; DD=D-dimer; F=females; EB=Epstein-Barr; EBV-VCA=Epstein Barr Virus-Viral Capsid Antigen; Hb=hemoglobin; FII=factor II; FVII=factor VII; FX=Factor XHtc=hematocrit; HS 1=HerpesSimplexType 1; M=males; MCHC=Mean Corpuscular Hemoglobin Concentration; Myc=Mycoplasma; NLR=neutrophil-to-lymphocyte ratio; PLT=platelets; PLR=platelets-to-lymphocytes ratio; PS=protein S; PT=prothrombin time; RBC=red blood cells; RDW=Red blood cells Distribution Width; Sch=Schistosoma; Var=Varicella.

Differences are calculated by Mann-Whitney U-test; statistically significant differences (p<0.05) are in bold.

Differences in terms of MRI metrics between relapsing- and remitting patients are reported in Supplementary Table 5.

Supplementary Table 5. Radiological (MRI) metrics in the relapsing- and remitting-multiple sclerosis patients' groups

		Median	IQR	*P*-value
**Lesion analyses**				
**FLAIR lesion volume (ml)**	Remitting	4.018	9.064	0.429
	Relapsing	5.573	6.688	
**N° of FLAIR lesions**	Remitting	31	30	0.879
	Relapsing	28.5	30.25	
**T1 lesion volume (ml)**	Remitting	2.237	5.092	0.49
	Relapsing	2.766	3.409	
**N° of T1 lesions**	Remitting	23	24	1
	Relapsing	21.5	24.25	
**Active (Gd+) lesion volume (ml)**	Remitting	0	0	**<0.001***
	Relapsing	0.021	0.195	
**N° of active (Gd+) lesions**	Remitting	0	0	**<0.001***
	Relapsing	1	1	
**Lesion perfusion metrics**				
**FLAIR LESIONS**				
**MTT FLAIR lesions**	Remitting	5.727	0.61	0.496
	Relapsing	5.911	1.102	
**MTT leakage* FLAIR lesions**	Remitting	5.496	0.537	0.692
	Relapsing	5.542	1.041	
**CBV FLAIR lesions**	Remitting	13.873	3.985	0.322
	Relapsing	11.504	3.753	
**CBV leakage* FLAIR lesions**	Remitting	13.53	8.054	0.361
	Relapsing	10.954	3.731	
**CBF FLAIR lesions**	Remitting	133.592	60.583	0.172
	Relapsing	124.459	26.469	
**CBF leakage* FLAIR lesions**	Remitting	134.468	53.225	0.175
	Relapsing	124.982	29.539	
**ACTIVE (GD+) LESIONS**				
**MTT active (Gd+) lesions**	Remitting	4.192	0.328	0.522
	Relapsing	6.136	2.968	
**MTT leakage* active (Gd+) lesions**	Remitting	4.192	0.328	0.302
	Relapsing	5.842	2.057	
**CBV active (Gd+) lesions**	Remitting	16.182	3.881	0.571
	Relapsing	15.268	7.914	
	Remitting	15.773	3.411	0.7

**CBV leakage* active (Gd+) lesions**	Relapsing	14.624	7.98	
**CBF active (Gd+) lesions**	Remitting	237.624	73.894	0.106
	Relapsing	138.681	23.385	
**CBF leakage* active (Gd+) lesions**	Remitting	230.98	67.23	0.131
	Relapsing	136.807	47.851	
**Normal appearing white matter (NAWM) perfusion metrics**				
**MTT NAWM**	Remitting	5.57	1.43	0.398
	Relapsing	5.323	0.886	
**MTT leakage NAWM**	Remitting	5.286	1.27	0.846
	Relapsing	5.299	0.962	
**CBV NAWM**	Remitting	14.994	3.786	**0.047***
	Relapsing	12.766	3.673	
**CBV leakage NAWM**	Remitting	13.178	3.891	0.129
	Relapsing	12.348	2.496	
**CBF NAWM**	Remitting	154.698	51.354	0.099
	Relapsing	140.974	49.49	
**CBF leakage NAWM**	Remitting	153.449	51.794	0.088
	Relapsing	140.738	48.514	
**Caudate perfusion metrics**				
**RIGHT (R-) CAUDATE**				
**MTT R-caudate**	Remitting	5.594	1.243	0.293
	Relapsing	5.835	1.234	
**MTT leakage* R-caudate**	Remitting	5.249	1.197	0.147
	Relapsing	5.751	1.078	
**CBV R-caudate**	Remitting	20.397	10.458	0.246
	Relapsing	18.84	6.08	
**CBV leakage* R-caudate**	Remitting	19.37	4.301	0.685
	Relapsing	18.871	5.288	
**CBF R-caudate**	Remitting	207.29	37.807	0.178
	Relapsing	197.017	54.449	
**CBF leakage* R-caudate**	Remitting	206.227	40.367	0.246
	Relapsing	200.48	48.808	
**LEFT (L-) CAUDATE**				
**MTT L-caudate**	Remitting	6.066	2.002	0.317
	Relapsing	6.567	1.889	
**MTT leakage* L-caudate**	Remitting	5.416	2.035	0.658
	Relapsing	5.593	1.447	
**CBV L-caudate**	Remitting	19.07	14.77	**0.044***
	Relapsing	16.354	7.597	
**CBV leakage* L-caudate**	Remitting	17.188	7.112	0.48
	Relapsing	16.936	6.403	
**CBF L-caudate**	Remitting	188.196	46.648	0.231
	Relapsing	172.806	66.508	
**CBF leakage* L-caudate**	Remitting	192.288	44.145	0.27
	Relapsing	172.929	57.527	

**GLOBAL CAUDATE**				
**MTT caudate**	Remitting	6.072	1.411	0.348
	Relapsing	6.298	1.393	
**MTT leakage* caudate**	Remitting	5.062	1.429	0.231
	Relapsing	5.591	1.116	
**CBV caudate**	Remitting	19.071	8.381	0.178
	Relapsing	16.125	5.098	
**CBV leakage* caudate**	Remitting	18.299	5.354	0.685
	Relapsing	18.316	5.226	
**CBF caudate**	Remitting	200.591	51.113	0.228
	Relapsing	189.019	52.784	
**CBF leakage* caudate**	Remitting	204.331	48.564	0.246
	Relapsing	190.769	45.443	
**Thalamus perfusion metrics**				
**RIGHT (R-) THALAMUS**				
**MTT R-thalamus**	Remitting	4.894	1.07	0.65
	Relapsing	4.821	0.767	
**MTT leakage* R-thalamus**	Remitting	4.894	1.248	0.468
	Relapsing	5.002	0.778	
**CBV R-thalamus**	Remitting	20.784	10.012	0.122
	Relapsing	16.649	4.947	
**CBV leakage* R-thalamus**	Remitting	19.144	5.502	0.381
	Relapsing	17.46	4.598	
**CBF R-thalamus**	Remitting	221.435	64.725	0.144
	Relapsing	202.024	54.967	
**CBF leakage* R-thalamus**	Remitting	220.05	68.056	0.224
	Relapsing	206.132	43.899	
**LEFT (L-) THALAMUS**				
**MTT L-thalamus**	Remitting	4.922	1.027	0.893
	Relapsing	4.878	0.762	
**MTT leakage* L-thalamus**	Remitting	4.983	1.081	0.843
	Relapsing	5.004	0.853	
**CBV L-thalamus**	Remitting	18.27	5.855	0.371
	Relapsing	16.628	5.314	
**CBV leakage* L-thalamus**	Remitting	19.911	4.649	0.246
	Relapsing	18.218	5.3	
**CBF L-thalamus**	Remitting	243.271	60.798	0.262
	Relapsing	207.982	50.358	
**CBF leakage* L-thalamus**	Remitting	240.425	54.105	0.337
	Relapsing	209.138	44.846	
**GLOBAL THALAMUS**				
**MTT thalamus**	Remitting	4.893	1.17	0.959
	Relapsing	4.931	0.649	
**MTT leakage* thalamus**	Remitting	4.939	1.001	0.943
	Relapsing	5.003	0.903	
**CBV thalamus**	Remitting	19.345	5.919	0.129

	Relapsing	16.631	4.596	
**CBV leakage* thalamus**	Remitting	19.528	4.621	0.226
	Relapsing	17.839	5.311	
**CBF thalamus**	Remitting	233.631	57.071	0.166
	Relapsing	204.775	50.485	
**CBF leakage* thalamus**	Remitting	232.044	57.092	0.197
	Relapsing	206.665	37.665	
**Globus pallidus perfusion metrics**				
**RIGHT (R-) GLOBUS PALLIDUS**				
**MTT R-globus pallidus**	Remitting	4.65	0.948	0.177
	Relapsing	5.037	0.889	
**MTT leakage* R-globus pallidus**	Remitting	4.681	0.912	0.371
	Relapsing	4.808	1.025	
**CBV R-globus pallidus**	Remitting	14.565	1.671	0.727
	Relapsing	15.381	4.792	
**CBV leakage* R-globus pallidus**	Remitting	14.218	2.518	0.749
	Relapsing	14.426	4.749	
**CBF R-globus pallidus**	Remitting	186.183	37.836	0.522
	Relapsing	170.683	67.317	
**CBF leakage* R-globus pallidus**	Remitting	185.686	42.993	0.651
	Relapsing	170.092	55.73	
**LEFT (L-) GLOBUS PALLIDUS**				
**MTT L-globus pallidus**	Remitting	4.682	1.019	0.24
	Relapsing	5.045	1.012	
**MTT leakage* L-globus pallidus**	Remitting	4.649	0.941	0.401
	Relapsing	4.839	0.91	
**CBV L-globus pallidus**	Remitting	14.847	3.351	0.644
	Relapsing	15.37	4.965	
**CBV leakage* L-globus pallidus**	Remitting	14.325	3.356	0.947
	Relapsing	13.862	3.948	
**CBF L-globus pallidus**	Remitting	183.80	30.351	0.468
	Relapsing	172.719	70.4	
**CBF leakage* L-globus pallidus**	Remitting	187.223	36.663	0.578
	Relapsing	172.798	69.284	
**GLOBAL GLOBUS PALLIDUS**				
**MTT globus pallidus**	Remitting	4.818	1.183	0.695
	Relapsing	5.008	0.939	
**MTT leakage* globus pallidus**	Remitting	4.778	0.835	0.304
	Relapsing	5.048	1.016	
**CBV globus pallidus**	Remitting	15.659	2.98	0.224
	Relapsing	14.636	4.008	
**CBV leakage* globus pallidus**	Remitting	14.65	4.205	0.54
	Relapsing	14.256	4.129	
**CBF globus pallidus**	Remitting	184.991	30.151	0.268
	Relapsing	171.701	70.200	
	Remitting	191.419	45.05	0.231

**CBF leakage* globus pallidus**	Relapsing	168.645	40.836	
**Putamen perfusion metrics**				
**RIGHT (R-) PUTAMEN**				
**MTT R-putamen**	Remitting	4.801	1.065	0.821
	Relapsing	4.794	0.991	
**MTT leakage* R-putamen**	Remitting	4.798	1.235	0.486
	Relapsing	4.765	1.085	
**CBV R-putamen**	Remitting	16.856	1.879	0.785
	Relapsing	16.981	4.022	
**CBV leakage* R-putamen**	Remitting	20.511	4.161	0.217
	Relapsing	19.568	4.886	
**CBF R-putamen**	Remitting	241.971	51.901	**0.037***
	Relapsing	222.19	44.211	
**CBF leakage* R-putamen**	Remitting	240.525	54.948	0.069
	Relapsing	222.099	44.724	
**LEFT (L-) PUTAMEN**				
**MTT L-putamen**	Remitting	4.824	1.273	0.359
	Relapsing	4.877	0.764	
**MTT leakage* L-putamen**	Remitting	4.775	1.29	0.371
	Relapsing	4.903	0.825	
**CBV L-putamen**	Remitting	17.812	5.065	0.197
	Relapsing	16.722	3.819	
**CBV leakage* L-putamen**	Remitting	19.916	4.513	0.149
	Relapsing	18.451	5.323	
**CBF L-putamen**	Remitting	243.1	68.018	**0.028***
	Relapsing	218.517	45.979	
**CBF leakage* L-putamen**	Remitting	245.098	71.643	0.093
	Relapsing	225.901	49.75	
**GLOBAL PUTAMEN**				
**MTT putamen**	Remitting	4.813	1.298	0.523
	Relapsing	4.81	0.883	
**MTT leakage* putamen**	Remitting	4.787	1.217	0.391
	Relapsing	4.885	0.883	
**CBV putamen**	Remitting	17.452	3.417	0.888
	Relapsing	17.396	3.847	
**CBV leakage* putamen**	Remitting	19.961	3.449	0.356
	Relapsing	19.115	4.755	
**CBF putamen**	Remitting	241.265	65.233	0.088
	Relapsing	223.309	52.828	
**CBF leakage* putamen**	Remitting	243.357	65.537	0.178
	Relapsing	226.166	45.974	

CBF=cerebral blood flow; CBV=cerebral blood volume; FLAIR=fluid-attenuated inversion recovery; Gd+=gadolinium enhancing; L=left; MTT=mean transit time; NAWM=normal-appearing white matter; R=right.
* leakage correction analysis (corrected for contrast agent extravasation).
Differences were calculated by Mann-Whitney test. Statistically significant p-values are reported in bold.

Correlations between patients' demographic/clinical features, laboratory and MRI data with rho>0.5 and p<0.05 are shown in Supplementary Table 6.

Supplementary Table 6. Correlations (Spearman's rho ≥ 0.5, *P*-value < 0.05) between demographic/clinical characteristics, laboratory biomarkers and radiological (MRI) metrics.

Variable 1	Variable 2	Spearman's rho	*P*-value
Correlations between demographics/clinical characteristics			
Age	Age at onset	0.649	<0.0001
Number of relapses in the previous 24 months	Number of relapses in the previous 12 months	0.915	<0.0001
Total number of relapses	EDSS at baseline	0.654	<0.0001
Z_SCORE_9-HPT	MSFC	0.858	<0.0001
Z_SCORE_PASAT_3"	MSFC	0.805	<0.0001
EDSS at baseline	Z_SCORE_9-HPT	-0.594	<0.0001
EDSS at baseline	MSFC	-0.571	<0.0001
Correlations between laboratory biomarkers			
Anti-Beta2_GPI_IgG	Anti-Beta2_GPI_IgM	0.560	<0.0001
Anti-cardiolipin GPI_IgG	Anti-Beta2_GPI_IgG	0.608	<0.0001
Anti-cardiolipin_IgM	Anti-Beta2_GPI_IgG	0.516	<0.0001
Anti-cardiolipin_IgM	Anti-Beta2_GPI_IgM	0.872	<0.0001
APTT	vWF	-0.535	<0.0001
APTT	aPTT-ratio	0.982	<0.0001
C3	FX	0.685	<0.0001
Chlamydia_IgG	Chlamydia IgA	0.726	<0.0001
Creatinin	eGFR	-0.751	<0.0001
Creatinin	Vitamin D	0.550	0.0029
EBV-VCA_IgM	Mycoplasma_IgM	0.531	<0.0001
EBV-VCA_IgM	HerpesSimplexType1_IgM	0.694	<0.0001
Epstein-Barr_IgM	HerpesSimplexType1_IgM	0.527	<0.0001
FIB	C3	0.563	<0.0001
FII	C3	0.507	<0.0001
FII	FVII	0.522	<0.0001
FVIII	vWF	0.845	<0.0001
WBC	Neu	0.862	<0.0001
RBC	Hb	0.650	<0.0001
RBC	Htc	0.710	<0.0001
Hb	Htc	0.964	<0.0001
HerpesSimplexType1_IgM	Cytomegalovirus_IgM	0.571	<0.0001
HerpesSimplexType1-2_IgG	HerpesSimplexType1_IgG	0.886	<0.0001
HerpesSimplexType1-2_IgM	HerpesSimplexType2_IgM	0.901	<0.0001
InfluenzaA_IgM	InfluenzaB_IgM	0.846	<0.0001
MCHC	RDW	-0.560	<0.0001
MCV	MHC	0.895	<0.0001
MHC	RDW	-0.573	<0.0001
MHC	MCHC	0.543	<0.0001
MPV	PDW	0.586	<0.0001
PC	FX	0.530	<0.0001
PC	FVII	0.553	<0.0001

PLD	PDW	-0.585	<0.0001
PLD	MPV	-0.509	<0.0001
PT	FII	-0.590	<0.0001
PT	FVII	-0.527	<0.0001
PT%	PT	-0.999	<0.0001
PT%	FVII	0.524	<0.0001
PT%	FII	0.596	<0.0001
T-CD3	B-CD19	0.575	<0.0001
T-CD3	T-CD8	0.830	<0.0001
T-CD3	T-CD4	0.902	<0.0001
T-CD4	T-CD8	0.559	<0.0001
T-CD4	B-CD19	0.607	<0.0001
T-CD8	CD4\CD8	-0.587	<0.0001
Correlations between radiological metrics			
FLAIR lesion load	number of lesions in FLAIR	0.717	<0.0001
FLAIR lesion load	number of lesions in T1	0.804	<0.0001
FLAIR lesion load	volume of lesions in T1	0.928	<0.0001
MTT leakage* caudate	MTT leakage* Rglobus pallidus	0.525	0.0001
MTT leakage* caudate	MTT leakage* FLAIR	0.582	<0.0001
MTT leakage* caudate	MTT leakage* globus pallidus	0.804	<0.0001
MTT leakage* caudate	MTT leakage* Rthalamus	0.835	<0.0001
MTT leakage* caudate	MTT leakage* Lthalamus	0.837	<0.0001
MTT leakage* caudate	MTT leakage* Lcaudate	0.842	<0.0001
MTT leakage* caudate	MTT leakage* Rcaudate	0.855	<0.0001
MTT leakage* caudate	MTT leakage* Lputamen	0.858	<0.0001
MTT leakage* caudate	MTT leakage* Rputamen	0.879	<0.0001
MTT leakage* caudate	MTT leakage* putamen	0.917	<0.0001
MTT leakage* FLAIR	rCBV leakage* FLAIR	0.502	0.0002
MTT leakage* FLAIR	MTT leakage* active (Gd+) lesions	0.602	0.0135
MTT leakage* Lcaudate	MTT leakage_re_Lglobus pallidus	0.534	0.0001
MTT leakage* Lcaudate	MTT leakage* active (Gd+) lesions	0.576	0.0196
MTT leakage* Lcaudate	MTT leakage_re_Rglobus pallidus	0.587	<0.0001
MTT leakage* Lcaudate	MTT leakage_re_FLAIR	0.622	<0.0001
MTT leakage* active (Gd+) lesions	rCBV leakage* active (Gd+) lesions	0.637	0.0079
MTT leakage* active (Gd+) lesions	rCBV leakage* active (Gd+) lesions	0.637	0.0079
MTT leakage* Lglobus pallidus	MTT leakage* active (Gd+) lesions	0.535	0.0329
MTT leakage* Lglobus pallidus	MTT leakage* FLAIR	0.622	<0.0001
MTT leakage* Lputamen	MTT leakage* Rglobus pallidus	0.529	0.0001
MTT leakage* Lputamen	MTT leakage* Lcaudate	0.714	<0.0001
MTT leakage* Lputamen	MTT leakage* Rcaudate	0.750	<0.0001
MTT leakage* Lputamen	MTT leakage* Lthalamus	0.878	<0.0001
MTT leakage* Lputamen	MTT leakage* Rthalamus	0.889	<0.0001
MTT leakage* Lthalamus	MTT leakage* Lglobus pallidus	0.505	0.0002
MTT leakage* Lthalamus	MTT leakage* Rglobus pallidus	0.591	<0.0001
MTT leakage* Lthalamus	MTT leakage* Lcaudate	0.736	<0.0001
MTT leakage* Lthalamus	MTT leakage* Rcaudate	0.773	<0.0001
MTT leakage* normal-appearing white matter	MTT leakage* Lglobus pallidus	0.560	<0.0001
MTT leakage* normal-appearing white matter	MTT leakage* FLAIR	0.570	<0.0001
MTT leakage* normal-appearing white matter	MTT leakage* active (Gd+) lesions	0.599	0.0142
MTT leakage* normal-appearing white matter	MTT leakage* Rglobus pallidus	0.672	<0.0001
MTT leakage* normal-appearing white matter	MTT leakage* Rcaudate	0.701	<0.0001
MTT leakage* normal-appearing white matter	MTT leakage* Rthalamus	0.745	<0.0001
MTT leakage* normal-appearing white matter	MTT leakage* Lputamen	0.774	<0.0001

MTT leakage* normal-appearing white matter	MTT leakage* caudate	0.800	<0.0001
MTT leakage* normal-appearing white matter	MTT leakage* Rputamen	0.809	<0.0001
MTT leakage* normal-appearing white matter	MTT leakage* putamen	0.823	<0.0001
MTT leakage* normal-appearing white matter	MTT leakage* Lcaudate	0.824	<0.0001
MTT leakage* normal-appearing white matter	MTT leakage* Lthalamus	0.832	<0.0001
MTT leakage* normal-appearing white matter	MTT leakage* globus pallidus	0.855	<0.0001
MTT leakage* globus pallidus	MTT leakage* FLAIR	0.512	0.0001
MTT leakage* globus pallidus	MTT leakage* active (Gd+) lesions	0.632	0.0087
MTT leakage* globus pallidus	MTT leakage* Lglobus pallidus	0.668	<0.0001
MTT leakage* globus pallidus	MTT leakage* Rcaudate	0.728	<0.0001
MTT leakage* globus pallidus	MTT leakage* Lcaudate	0.735	<0.0001
MTT leakage* globus pallidus	MTT leakage* Lputamen	0.762	<0.0001
MTT leakage* globus pallidus	MTT leakage* Rglobus pallidus	0.767	<0.0001
MTT leakage* globus pallidus	MTT leakage* Rthalamus	0.806	<0.0001
MTT leakage* globus pallidus	MTT leakage* Lthalamus	0.847	<0.0001
MTT leakage* globus pallidus	MTT leakage* putamen	0.865	<0.0001
MTT leakage* globus pallidus	MTT leakage* Rputamen	0.881	<0.0001
MTT leakage* putamen	MTT leakage* Lglobus pallidus	0.502	0.0002
MTT leakage* putamen	MTT leakage* FLAIR	0.537	<0.0001
MTT leakage* putamen	MTT leakage* active (Gd+) lesions	0.549	0.0275
MTT leakage* putamen	MTT leakage* Rglobus pallidus	0.627	<0.0001
MTT leakage* putamen	MTT leakage* Rcaudate	0.796	<0.0001
MTT leakage* putamen	MTT leakage* Lcaudate	0.804	<0.0001
MTT leakage* putamen	MTT leakage* Lthalamus	0.924	<0.0001
MTT leakage* putamen	MTT leakage* Rthalamus	0.929	<0.0001
MTT leakage* putamen	MTT leakage* Lputamen	0.948	<0.0001
MTT leakage* putamen	MTT leakage* Rputamen	0.954	<0.0001
MTT leakage* Rcaudate	MTT leakage* Lglobus pallidus	0.581	<0.0001
MTT leakage* Rcaudate	MTT leakage* Rglobus pallidus	0.583	<0.0001
MTT leakage* Rcaudate	MTT leakage* FLAIR	0.645	<0.0001
MTT leakage* Rcaudate	MTT leakage* Lcaudate	0.731	<0.0001
MTT leakage* Rglobus pallidus	MTT leakage* FLAIR	0.505	0.0002
MTT leakage* Rglobus pallidus	MTT leakage* active (Gd+) lesions	0.605	0.0131
MTT leakage* Rglobus pallidus	MTT leakage* Lglobus pallidus	0.837	<0.0001
MTT leakage* Rputamen	MTT leakage* Lglobus pallidus	0.534	0.0001
MTT leakage* Rputamen	MTT leakage* Rglobus pallidus	0.658	<0.0001
MTT leakage* Rputamen	MTT leakage* active (Gd+) lesions	0.658	0.0056
MTT leakage* Rputamen	MTT leakage* Rcaudate	0.732	<0.0001
MTT leakage* Rputamen	MTT leakage* Lcaudate	0.793	<0.0001
MTT leakage* Rputamen	MTT leakage* Lputamen	0.819	<0.0001
MTT leakage* Rputamen	MTT leakage* Lthalamus	0.884	<0.0001
MTT leakage* Rputamen	MTT leakage* Rthalamus	0.889	<0.0001
MTT leakage* Rthalamus	MTT leakage* Rglobus pallidus	0.600	<0.0001
MTT leakage* Rthalamus	MTT leakage* Lcaudate	0.703	<0.0001
MTT leakage* Rthalamus	MTT leakage* Rcaudate	0.737	<0.0001
MTT leakage* Rthalamus	MTT leakage* Lthalamus	0.920	<0.0001
number of lesions in FLAIR	volume of lesions in T1	0.699	<0.0001
number of lesions in FLAIR	number of lesions in T1	0.914	<0.0001
rCBF leakage* caudate	rCBV leakage* Lthalamus	0.533	0.0001
rCBF leakage* caudate	rCBV leakage* Rglobus pallidus	0.541	<0.0001
rCBF leakage* caudate	rCBV leakage* Lputamen	0.551	<0.0001
rCBF leakage* caudate	rCBV leakage* Rthalamus	0.553	<0.0001
rCBF leakage* caudate	rCBF leakage* FLAIR	0.557	<0.0001
rCBF leakage* caudate	rCBF leakage* Lglobus pallidus	0.595	<0.0001

rCBF leakage* caudate	rCBV leakage* globus pallidus	0.603	<0.0001
rCBF leakage* caudate	rCBV leakage* putamen	0.618	<0.0001
rCBF leakage* caudate	rCBV leakage* Rputamen	0.631	<0.0001
rCBF leakage* caudate	rCBV leakage* Lcaudate	0.693	<0.0001
rCBF leakage* caudate	rCBV leakage* Rcaudate	0.714	<0.0001
rCBF leakage* caudate	rCBF leakage* Rglobus pallidus	0.723	<0.0001
rCBF leakage* caudate	rCBF leakage* Lthalamus	0.738	<0.0001
rCBF leakage* caudate	rCBF leakage* Rthalamus	0.771	<0.0001
rCBF leakage* caudate	rCBF leakage* thalamus	0.803	<0.0001
rCBF leakage* caudate	rCBF leakage* Rputamen	0.815	<0.0001
rCBF leakage* caudate	rCBF leakage* globus pallidus	0.858	<0.0001
rCBF leakage* caudate	rCBF leakage* Lputamen	0.895	<0.0001
rCBF leakage* caudate	rCBF leakage* putamen	0.908	<0.0001
rCBF leakage* caudate	rCBF leakage* Rcaudate	0.927	<0.0001
rCBF leakage* caudate	rCBF leakage* Lcaudate	0.929	<0.0001
rCBF leakage* FLAIR	rCBF leakage* active (Gd+) lesions	0.543	0.0297
rCBF leakage* FLAIR	rCBV leakage* active (Gd+) lesions	0.597	0.0146
rCBF leakage* L caudate	rCBV leakage* active (Gd+) lesions	0.528	0.0354
rCBF leakage* L caudate	rCBF leakage* FLAIR	0.547	<0.0001
rCBF leakage* L caudate	rCBV leakage* Rglobus pallidus	0.612	<0.0001
rCBF leakage* L caudate	rCBF leakage* Lglobus pallidus	0.639	<0.0001
rCBF leakage* L caudate	rCBF leakage* Rglobus pallidus	0.760	<0.0001
rCBF leakage* Lglobus pallidus	rCBF leakage* active (Gd+) lesions	0.611	0.0119
rCBF leakage* Lglobus pallidus	rCBV leakage* FLAIR	0.663	<0.0001
rCBF leakage* Lglobus pallidus	rCBV leakage* active (Gd+) lesions	0.670	0.0045
rCBF leakage* Lglobus pallidus	rCBF leakage* FLAIR	0.746	<0.0001
rCBF leakage* Lputamen	rCBF leakage* FLAIR	0.578	<0.0001
rCBF leakage* Lputamen	rCBV leakage* Lcaudate	0.635	<0.0001
rCBF leakage* Lputamen	rCBV leakage* Rcaudate	0.643	<0.0001
rCBF leakage* Lputamen	rCBV leakage* Lthalamus	0.664	<0.0001
rCBF leakage* Lputamen	rCBF leakage* Lglobus pallidus	0.665	<0.0001
rCBF leakage* Lputamen	rCBV leakage* Rthalamus	0.669	<0.0001
rCBF leakage* Lputamen	rCBF leakage* Rglobus pallidus	0.767	<0.0001
rCBF leakage* Lputamen	rCBF leakage* Lcaudate	0.805	<0.0001
rCBF leakage* Lputamen	rCBF leakage* Rcaudate	0.845	<0.0001
rCBF leakage* Lputamen	rCBF leakage* Lthalamus	0.856	<0.0001
rCBF leakage* Lputamen	rCBF leakage* Rthalamus	0.921	<0.0001
rCBF leakage* Lthalamus	rCBF leakage* FLAIR	0.559	<0.0001
rCBF leakage* Lthalamus	rCBV leakage* Rcaudate	0.580	<0.0001
rCBF leakage* Lthalamus	rCBV leakage* Lcaudate	0.583	<0.0001
rCBF leakage* Lthalamus	rCBF leakage* Lglobus pallidus	0.690	<0.0001
rCBF leakage* Lthalamus	rCBF leakage* Lcaudate	0.746	<0.0001
rCBF leakage* Lthalamus	rCBF leakage* Rglobus pallidus	0.747	<0.0001
rCBF leakage* Lthalamus	rCBF leakage* Rcaudate	0.796	<0.0001
rCBF leakage* normal-appearing white matter	rCBV leakage* Lputamen	0.507	0.0001
rCBF leakage* normal-appearing white matter	rCBF leakage* FLAIR	0.527	0.0001
rCBF leakage* normal-appearing white matter	rCBV leakage* active (Gd+) lesions	0.582	0.0179
rCBF leakage* normal-appearing white matter	rCBV leakage* putamen	0.584	<0.0001
rCBF leakage* normal-appearing white matter	rCBV leakage* Rputamen	0.588	<0.0001
rCBF leakage* normal-appearing white matter	rCBV leakage* Rcaudate	0.593	<0.0001
rCBF leakage* normal-appearing white matter	rCBV leakage* Lcaudate	0.602	<0.0001
rCBF leakage* normal-appearing white matter	rCBV leakage* globus pallidus	0.604	<0.0001
rCBF leakage* normal-appearing white matter	rCBF leakage* active (Gd+) lesions	0.623	0.01
rCBF leakage* normal-appearing white matter	rCBV leakage* caudate nuleus	0.629	<0.0001

rCBF leakage* normal-appearing white matter	rCBV leakage* Lthalamus	0.649	<0.0001
rCBF leakage* normal-appearing white matter	rCBF leakage* Lglobus pallidus	0.659	<0.0001
rCBF leakage* normal-appearing white matter	rCBV leakage* Rthalamus	0.673	<0.0001
rCBF leakage* normal-appearing white matter	rCBF leakage* Rglobus pallidus	0.695	<0.0001
rCBF leakage* normal-appearing white matter	rCBF leakage* Rputamen	0.784	<0.0001
rCBF leakage* normal-appearing white matter	rCBF leakage* Rcaudate	0.791	<0.0001
rCBF leakage* normal-appearing white matter	rCBF leakage* Lcaudate	0.812	<0.0001
rCBF leakage* normal-appearing white matter	rCBF leakage* Lthalamus	0.824	<0.0001
rCBF leakage* normal-appearing white matter	rCBF leakage* caudate	0.843	<0.0001
rCBF leakage* normal-appearing white matter	rCBF leakage* Rthalamus	0.875	<0.0001
rCBF leakage* normal-appearing white matter	rCBF leakage* Lputamen	0.884	<0.0001
rCBF leakage* normal-appearing white matter	rCBF leakage* putamen	0.889	<0.0001
rCBF leakage* normal-appearing white matter	rCBF leakage* globus pallidus	0.892	<0.0001
rCBF leakage* normal-appearing white matter	rCBF leakage* thalamus	0.910	<0.0001
rCBF leakage* globus pallidus	rCBV leakage* Lglobus pallidus	0.531	0.0001
rCBF leakage* globus pallidus	rCBF leakage* active (Gd+) lesions	0.543	0.0297
rCBF leakage* globus pallidus	rCBV leakage* active (Gd+) lesions	0.571	0.021
rCBF leakage* globus pallidus	rCBV leakage* Lputamen	0.572	<0.0001
rCBF leakage* globus pallidus	rCBV leakage* Rglobus pallidus	0.574	<0.0001
rCBF leakage* globus pallidus	rCBV leakage* Rcaudate	0.596	<0.0001
rCBF leakage* globus pallidus	rCBF leakage* FLAIR	0.643	<0.0001
rCBF leakage* globus pallidus	rCBV leakage* Rputamen	0.651	<0.0001
rCBF leakage* globus pallidus	rCBV leakage* putamen	0.652	<0.0001
rCBF leakage* globus pallidus	rCBV leakage* Lcaudate	0.664	<0.0001
rCBF leakage* globus pallidus	rCBV leakage* Rthalamus	0.665	<0.0001
rCBF leakage* globus pallidus	rCBV leakage* Lthalamus	0.673	<0.0001
rCBF leakage* globus pallidus	rCBF leakage* Lglobus pallidus	0.766	<0.0001
rCBF leakage* globus pallidus	rCBF leakage* Rcaudate	0.818	<0.0001
rCBF leakage* globus pallidus	rCBF leakage* Lcaudate	0.824	<0.0001
rCBF leakage* globus pallidus	rCBF leakage* Rputamen	0.834	<0.0001
rCBF leakage* globus pallidus	rCBF leakage* Rglobus pallidus	0.834	<0.0001
rCBF leakage* globus pallidus	rCBF leakage* Lthalamus	0.889	<0.0001
rCBF leakage* globus pallidus	rCBF leakage* Rthalamus	0.918	<0.0001
rCBF leakage* globus pallidus	rCBF leakage* Lputamen	0.921	<0.0001
rCBF leakage* globus pallidus	rCBF leakage* putamen	0.932	<0.0001
rCBF leakage* putamen	rCBV leakage* active (Gd+) lesions	0.511	0.0432
rCBF leakage* putamen	rCBV leakage* Rglobus pallidus	0.515	0.0001
rCBF leakage* putamen	rCBF leakage* FLAIR	0.597	<0.0001
rCBF leakage* putamen	rCBV leakage* Lputamen	0.620	<0.0001
rCBF leakage* putamen	rCBV leakage* Lcaudate	0.655	<0.0001
rCBF leakage* putamen	rCBV leakage* Rcaudate	0.661	<0.0001
rCBF leakage* putamen	rCBF leakage* Lglobus pallidus	0.674	<0.0001
rCBF leakage* putamen	rCBV leakage* Lthalamus	0.675	<0.0001
rCBF leakage* putamen	rCBV leakage* Rthalamus	0.677	<0.0001
rCBF leakage* putamen	rCBV leakage* Rputamen	0.687	<0.0001
rCBF leakage* putamen	rCBF leakage* Rglobus pallidus	0.782	<0.0001
rCBF leakage* putamen	rCBF leakage* Lcaudate	0.821	<0.0001
rCBF leakage* putamen	rCBF leakage* Lthalamus	0.870	<0.0001
rCBF leakage* putamen	rCBF leakage* Rcaudate	0.871	<0.0001
rCBF leakage* putamen	rCBF leakage* Rputamen	0.897	<0.0001
rCBF leakage* putamen	rCBF leakage* Rthalamus	0.930	<0.0001
rCBF leakage* putamen	rCBF leakage* Lputamen	0.992	<0.0001
rCBF leakage* Rcaudate	rCBF leakage* FLAIR	0.548	<0.0001
rCBF leakage* Rcaudate	rCBV leakage* active (Gd+) lesions	0.558	0.0248

rCBF leakage* Rcaudate	rCBF leakage* Lglobus pallidus	0.587	<0.0001
rCBF leakage* Rcaudate	rCBV leakage* Lcaudate nuclues	0.659	<0.0001
rCBF leakage* Rcaudate	rCBF leakage* Rglobus pallidus	0.724	<0.0001
rCBF leakage* Rcaudate	rCBF leakage* Lcaudate	0.891	<0.0001
rCBF leakage* Rglobus pallidus	rCBF leakage* FLAIR	0.549	<0.0001
rCBF leakage* Rglobus pallidus	rCBF leakage* Lglobus pallidus	0.793	<0.0001
rCBF leakage* Rputamen	rCBF leakage* FLAIR	0.521	0.0001
rCBF leakage* Rputamen	rCBV leakage* Rcaudate	0.551	<0.0001
rCBF leakage* Rputamen	rCBV leakage* Lcaudate	0.554	<0.0001
rCBF leakage* Rputamen	rCBV leakage* Lthalamus	0.559	<0.0001
rCBF leakage* Rputamen	rCBV leakage* Rthalamus	0.562	<0.0001
rCBF leakage* Rputamen	rCBF leakage* Lglobus pallidus	0.583	<0.0001
rCBF leakage* Rputamen	rCBF leakage* Rglobus pallidus	0.682	<0.0001
rCBF leakage* Rputamen	rCBV leakage* Lputamen	0.714	<0.0001
rCBF leakage* Rputamen	rCBF leakage* Lcaudate	0.717	<0.0001
rCBF leakage* Rputamen	rCBF leakage* Lthalamus	0.755	<0.0001
rCBF leakage* Rputamen	rCBF leakage* Rcaudate	0.766	<0.0001
rCBF leakage* Rputamen	rCBF leakage* Rthalamus	0.827	<0.0001
rCBF leakage* Rputamen	rCBF leakage* Lputamen	0.981	<0.0001
rCBF_leakage* Rthalamus	rCBV leakage* Lglobus pallidus	0.512	0.0001
rCBF_leakage* Rthalamus	rCBV leakage* Rcaudate	0.570	<0.0001
rCBF_leakage* Rthalamus	rCBV leakage* Lcaudate nucleus	0.583	<0.0001
rCBF_leakage* Rthalamus	rCBF leakage* FLAIR	0.606	<0.0001
rCBF_leakage* Rthalamus	rCBF leakage* Lcaudate	0.682	<0.0001
rCBF_leakage* Rthalamus	rCBF leakage* Lglobus pallidus	0.713	<0.0001
rCBF_leakage* Rthalamus	rCBV leakage* Lthalamus	0.722	<0.0001
rCBF_leakage* Rthalamus	rCBF leakage* Rglobus pallidus	0.741	<0.0001
rCBF_leakage* Rthalamus	rCBF leakage* Rcaudate	0.750	<0.0001
rCBF_leakage* Rthalamus	rCBF leakage* Lthalamus	0.885	<0.0001
rCBF leakage* thalamus	rCBV leakage* Rglobus pallidus	0.515	0.0001
rCBF leakage* thalamus	rCBV leakage* Lglobus pallidus	0.519	0.0001
rCBF leakage* thalamus	rCBV leakage* Lputamen	0.580	<0.0001
rCBF leakage* thalamus	rCBV leakage* Rcaudate nucleus	0.598	<0.0001
rCBF leakage* thalamus	rCBF leakage* FLAIR	0.605	<0.0001
rCBF leakage* thalamus	rCBV leakage* Lcaudate	0.619	<0.0001
rCBF leakage* thalamus	rCBV leakage* putamen	0.649	<0.0001
rCBF leakage* thalamus	rCBV leakage* Rputamen	0.659	<0.0001
rCBF leakage* thalamus	rCBV leakage* globus pallidus	0.677	<0.0001
rCBF leakage* thalamus	rCBF leakage* Lglobus pallidus	0.720	<0.0001
rCBF leakage* thalamus	rCBF leakage* Lcaudate	0.727	<0.0001
rCBF leakage* thalamus	rCBV leakage* Lthalamus	0.741	<0.0001
rCBF leakage* thalamus	rCBV leakage* Rthalamus	0.750	<0.0001
rCBF leakage* thalamus	rCBF leakage* Rglobus pallidus	0.760	<0.0001
rCBF leakage* thalamus	rCBF leakage* Rcaudate	0.766	<0.0001
rCBF leakage* thalamus	rCBF leakage* Rputamen	0.832	<0.0001
rCBF leakage* thalamus	rCBF leakage* Lthalamus	0.906	<0.0001
rCBF leakage* thalamus	rCBF leakage* globus pallidus	0.933	<0.0001
rCBF leakage* thalamus	rCBF leakage* Lputamen	0.939	<0.0001
rCBF leakage* thalamus	rCBF leakage* putamen	0.941	<0.0001
rCBF leakage* thalamus	rCBF leakage* Rthalamus	0.989	<0.0001
rCBV leakage* caudate	rCBV leakage* Lglobus pallidus	0.529	0.0001
rCBV leakage* caudate	rCBV leakage* active (Gd+) lesions	0.562	0.0235
rCBV leakage* caudate	rCBF leakage* Lthalamus	0.586	<0.0001
rCBV leakage* caudate	rCBF leakage* Rputamen	0.596	<0.0001

rCBV leakage* caudate	rCBF leakage* Rthalamus	0.612	<0.0001
rCBV leakage* caudate	rCBF leakage* Rglobus pallidus	0.613	<0.0001
rCBV leakage* caudate	rCBV leakage* FLAIR	0.630	<0.0001
rCBV leakage* caudate	rCBF leakage* FLAIR	0.641	<0.0001
rCBV leakage* caudate	rCBF leakage* thalamus	0.645	<0.0001
rCBV leakage* caudate	rCBF leakage* globus pallidus	0.659	<0.0001
rCBV leakage* caudate	rCBF leakage* Lputamen	0.681	<0.0001
rCBV leakage* caudate	rCBF leakage* putamen	0.698	<0.0001
rCBV leakage* caudate	rCBF leakage* Rcaudate	0.723	<0.0001
rCBV leakage* caudate	rCBV leakage* Rglobus pallidus	0.723	<0.0001
rCBV leakage* caudate	rCBF leakage* caudate	0.751	<0.0001
rCBV leakage* caudate	rCBF leakage* Lcaudate	0.762	<0.0001
rCBV leakage* caudate	rCBV leakage* Lputamen	0.787	<0.0001
rCBV leakage* caudate	rCBV leakage* Lthalamus	0.800	<0.0001
rCBV leakage* caudate	rCBV leakage* Rthalamus	0.825	<0.0001
rCBV leakage* caudate	rCBV leakage* globus pallidus	0.850	<0.0001
rCBV leakage* caudate	rCBV leakage* putamen	0.884	<0.0001
rCBV leakage* caudate	rCBV leakage* Rputamen	0.902	<0.0001
rCBV leakage* caudate	rCBV leakage* Rcaudate	0.950	<0.0001
rCBV leakage* caudate	rCBV leakage* Lcaudate	0.956	<0.0001
rCBV leakage* FLAIR	rCBF leakage* active (Gd+) lesions	0.584	0.0175
rCBV leakage* FLAIR	rCBV leakage* active (Gd+) lesions	0.665	0.005
rCBV leakage* FLAIR	rCBF leakage* FLAIR	0.936	<0.0001
rCBV leakage* Lcaudate	rCBF leakage* Lglobus pallidus	0.540	<0.0001
rCBV leakage* Lcaudate	rCBV leakage* Lglobus pallidus	0.584	<0.0001
rCBV leakage* Lcaudate	rCBF leakage* Rglobus pallidus	0.600	<0.0001
rCBV leakage* Lcaudate	rCBV leakage* FLAIR	0.640	<0.0001
rCBV leakage* Lcaudate	MTT leakage* active (Gd+) lesions	0.652	0.0062
rCBV leakage* Lcaudate	rCBV leakage* active (Gd+) lesions	0.658	0.0056
rCBV leakage* Lcaudate	rCBF leakage* FLAIR	0.658	<0.0001
rCBV leakage* Lcaudate	rCBV leakage* Rglobus pallidus	0.736	<0.0001
rCBV leakage* Lcaudate	rCBF leakage* Lcaudate	0.763	<0.0001
rCBV leakage* active (Gd+) lesions	rCBF leakage* active (Gd+) lesions	0.840	<0.0001
rCBV leakage* active (Gd+) lesions	rCBF leakage* active (Gd+) lesions	0.840	<0.0001
rCBV leakage* Lglobus pallidus	MTT leakage* active (Gd+) lesions	0.579	0.0188
rCBV leakage* Lglobus pallidus	rCBF leakage* FLAIR	0.612	<0.0001
rCBV leakage* Lglobus pallidus	rCBV leakage* FLAIR	0.672	<0.0001
rCBV leakage* Lglobus pallidus	rCBV leakage* active (Gd+) lesions	0.675	0.0041
rCBV leakage* Lglobus pallidus	rCBF leakage* Lglobus pallidus	0.699	<0.0001
rCBV leakage* Lputamen	rCBV leakage* FLAIR	0.508	0.0001
rCBV leakage* Lputamen	rCBF leakage* Lthalamus	0.515	0.0001
rCBV leakage* Lputamen	rCBF leakage* Rglobus pallidus	0.516	0.0001
rCBV leakage* Lputamen	rCBF leakage* FLAIR	0.523	0.0001
rCBV leakage* Lputamen	rCBF leakage* Rcaudate	0.535	0.0001
rCBV leakage* Lputamen	rCBF leakage* Lcaudate nculeus	0.550	<0.0001
rCBV leakage* Lputamen	rCBF leakage* Rthalamus	0.566	<0.0001
rCBV leakage* Lputamen	rCBV leakage* Rglobus pallidus	0.624	<0.0001
rCBV leakage* Lputamen	rCBF leakage* Lputamen	0.714	<0.0001
rCBV leakage* Lputamen	rCBV leakage* Rcaudate	0.745	<0.0001
rCBV leakage* Lputamen	rCBV leakage* Lthalamus	0.780	<0.0001
rCBV leakage* Lputamen	rCBV leakage* Lcaudate	0.781	<0.0001
rCBV leakage* Lputamen	rCBV leakage* Rthalamus	0.782	<0.0001
rCBV leakage* Lthalamus	rCBF leakage* Lglobus pallidus	0.536	0.0001
rCBV leakage* Lthalamus	rCBF leakage* Rglobus pallidus	0.547	<0.0001

rCBV leakage* Lthalamus	rCBF leakage* Lcaudate	0.549	<0.0001
rCBV leakage* Lthalamus	MTT leakage* active (Gd+) lesions	0.567	0.0221
rCBV leakage* Lthalamus	rCBV leakage* Lglobus pallidus	0.574	<0.0001
rCBV leakage* Lthalamus	rCBF leakage* Rcaudate nucleus	0.582	<0.0001
rCBV leakage* Lthalamus	rCBV leakage* FLAIR	0.585	<0.0001
rCBV leakage* Lthalamus	rCBF leakage* FLAIR	0.605	<0.0001
rCBV leakage* Lthalamus	rCBF leakage* active (Gd+) lesions	0.614	0.0115
rCBV leakage* Lthalamus	rCBV leakage* Rglobus pallidus	0.632	<0.0001
rCBV leakage* Lthalamus	rCBF leakage* Lthalamus	0.721	<0.0001
rCBV leakage* Lthalamus	rCBV leakage* active (Gd+) lesions	0.750	0.0008
rCBV leakage* Lthalamus	rCBV leakage* Rcaudate	0.787	<0.0001
rCBV leakage* Lthalamus	rCBV leakage* Lcaudate nuclleus	0.800	<0.0001
rCBV leakage* normal-appearing white matter	rCBF leakage* Rglobus pallidus	0.526	0.0001
rCBV leakage* normal-appearing white matter	rCBV leakage* FLAIR	0.539	<0.0001
rCBV leakage* normal-appearing white matter	rCBF leakage* FLAIR	0.540	<0.0001
rCBV leakage* normal-appearing white matter	rCBV leakage* Lglobus pallidus	0.562	<0.0001
rCBV leakage* normal-appearing white matter	rCBF leakage* Rputamen	0.571	<0.0001
rCBV leakage* normal-appearing white matter	rCBF leakage* Rcaudate	0.595	<0.0001
rCBV leakage* normal-appearing white matter	rCBF leakage* caudate	0.614	<0.0001
rCBV leakage* normal-appearing white matter	rCBF leakage* Lthalamus	0.623	<0.0001
rCBV leakage* normal-appearing white matter	rCBV leakage* active (Gd+) lesions	0.624	0.0099
rCBV leakage* normal-appearing white matter	rCBF leakage* Lcaudate	0.635	<0.0001
rCBV leakage* normal-appearing white matter	rCBV leakage* Rglobus pallidus	0.645	<0.0001
rCBV leakage* normal-appearing white matter	rCBF leakage* Lputamen	0.658	<0.0001
rCBV leakage* normal-appearing white matter	rCBF leakage* globus pallidus	0.659	<0.0001
rCBV leakage* normal-appearing white matter	rCBF leakage* putamen	0.671	<0.0001
rCBV leakage* normal-appearing white matter	rCBF leakage* Rthalamus	0.685	<0.0001
rCBV leakage* normal-appearing white matter	rCBF leakage* thalamus	0.719	<0.0001
rCBV leakage* normal-appearing white matter	rCBV leakage* Lputamen	0.736	<0.0001
rCBV leakage* normal-appearing white matter	rCBF leakage* normal-appearing white matter	0.770	<0.0001
rCBV leakage* normal-appearing white matter	rCBV leakage* Lcaudate	0.821	<0.0001
rCBV leakage* normal-appearing white matter	rCBV leakage* Rcaudate	0.823	<0.0001
rCBV leakage* normal-appearing white matter	rCBV leakage* globus pallidus	0.827	<0.0001
rCBV leakage* normal-appearing white matter	rCBV leakage* putamen	0.830	<0.0001
rCBV leakage* normal-appearing white matter	rCBV leakage* caudate	0.846	<0.0001
rCBV leakage* normal-appearing white matter	rCBV leakage* Rputamen	0.848	<0.0001
rCBV leakage* normal-appearing white matter	rCBV leakage* Lthalamus	0.879	<0.0001
rCBV leakage* normal-appearing white matter	rCBV leakage* Rthalamus	0.906	<0.0001
rCBV leakage* globus pallidus	rCBF leakage* Rcaudate	0.561	<0.0001
rCBV leakage* globus pallidus	rCBF leakage* Lglobus pallidus	0.569	<0.0001
rCBV leakage* globus pallidus	rCBF leakage* Rputamen	0.570	<0.0001
rCBV leakage* globus pallidus	rCBF leakage* Lthalamus	0.596	<0.0001
rCBV leakage* globus pallidus	rCBV leakage* FLAIR	0.611	<0.0001
rCBV leakage* globus pallidus	rCBF leakage* Lcaudate	0.621	<0.0001
rCBV leakage* globus pallidus	rCBF leakage* Rglobus pallidus	0.621	<0.0001
rCBV leakage* globus pallidus	rCBF leakage* FLAIR	0.626	<0.0001
rCBV leakage* globus pallidus	MTT leakage* active (Gd+) lesions	0.628	0.0091
rCBV leakage* globus pallidus	rCBF leakage* Lputamen	0.637	<0.0001
rCBV leakage* globus pallidus	rCBV leakage* active (Gd+) lesions	0.650	0.0064
rCBV leakage* globus pallidus	rCBF leakage* Rthalamus	0.656	<0.0001
rCBV leakage* globus pallidus	rCBF leakage* putamen	0.661	<0.0001
rCBV leakage* globus pallidus	rCBV leakage* Lglobus pallidus	0.681	<0.0001
rCBV leakage* globus pallidus	rCBF leakage* globus pallidus	0.715	<0.0001
rCBV leakage* globus pallidus	rCBV leakage* Rcaudate	0.754	<0.0001

rCBV leakage* globus pallidus	rCBV leakage* Lputamen	0.782	<0.0001
rCBV leakage* globus pallidus	rCBV leakage* Rglobus pallidus	0.805	<0.0001
rCBV leakage* globus pallidus	rCBV leakage* Rthalamus	0.845	<0.0001
rCBV leakage* globus pallidus	rCBV leakage* Lthalamus	0.855	<0.0001
rCBV leakage* globus pallidus	rCBV leakage* putamen	0.871	<0.0001
rCBV leakage* globus pallidus	rCBV leakage* Lcaudate	0.876	<0.0001
rCBV leakage* globus pallidus	rCBV leakage* Rputamen	0.908	<0.0001
rCBV leakage* putamen	rCBV leakage* Lglobus pallidus	0.523	0.0001
rCBV leakage* putamen	rCBV leakage* FLAIR	0.558	<0.0001
rCBV leakage* putamen	rCBF leakage* Rglobus pallidus	0.579	<0.0001
rCBV leakage* putamen	rCBF leakage* Rputamen	0.582	<0.0001
rCBV leakage* putamen	MTT leakage* active (Gd+) lesions	0.582	0.0181
rCBV leakage* putamen	rCBF leakage* FLAIR	0.598	<0.0001
rCBV leakage* putamen	rCBF leakage* Rthalamus	0.640	<0.0001
rCBV leakage* putamen	rCBF leakage* Lcaudate	0.660	<0.0001
rCBV leakage* putamen	rCBF leakage* Lthalamus	0.660	<0.0001
rCBV leakage* putamen	rCBF leakage_re_Rcaudate	0.663	<0.0001
rCBV leakage* putamen	rCBF leakage_re_Lputamen	0.673	<0.0001
rCBV leakage* putamen	rCBV leakage* active (Gd+) lesions	0.687	0.0033
rCBV leakage* putamen	rCBF leakage* putamen	0.690	<0.0001
rCBV leakage* putamen	rCBV leakage* Rglobus pallidus	0.711	<0.0001
rCBV leakage* putamen	rCBV leakage* Rcaudate	0.853	<0.0001
rCBV leakage* putamen	rCBV leakage* Lputamen	0.860	<0.0001
rCBV leakage* putamen	rCBV leakage* Rthalamus	0.876	<0.0001
rCBV leakage* putamen	rCBV leakage* Lcaudate	0.897	<0.0001
rCBV leakage* putamen	rCBV leakage* Lthalamus	0.898	<0.0001
rCBV leakage* putamen	rCBV leakage* Rputamen	0.963	<0.0001
rCBV leakage* Rcaudate	MTT leakage* active (Gd+) lesions	0.538	0.0318
rCBV leakage* Rcaudate	rCBF leakage* Rglobus pallidus	0.553	<0.0001
rCBV leakage* Rcaudate	rCBV leakage* FLAIR	0.569	<0.0001
rCBV leakage* Rcaudate	rCBF leakage* FLAIR	0.571	<0.0001
rCBV leakage* Rcaudate	rCBV leakage* Rglobus pallidus	0.653	<0.0001
rCBV leakage* Rcaudate	rCBV leakage* active (Gd+) lesions	0.655	0.0059
rCBV leakage* Rcaudate	rCBF leakage* Lcaudate	0.709	<0.0001
rCBV leakage* Rcaudate	rCBF leakage* Rcaudate	0.739	<0.0001
rCBV leakage* Rcaudate	rCBV leakage* Lcaudate	0.863	<0.0001
rCBV leakage* Rglobus pallidus	MTT leakage* active (Gd+) lesions	0.582	0.0181
rCBV leakage* Rglobus pallidus	rCBV leakage* active (Gd+) lesions	0.596	0.0148
rCBV leakage* Rglobus pallidus	rCBF leakage* Rglobus pallidus	0.670	<0.0001
rCBV leakage* Rglobus pallidus	rCBV leakage* Lglobus pallidus	0.686	<0.0001
rCBV leakage* Rputamen	MTT leakage* Lcaudate	0.501	0.0002
rCBV leakage* Rputamen	rCBV leakage* Lglobus pallidus	0.558	<0.0001
rCBV leakage* Rputamen	rCBF leakage* Rglobus pallidus	0.561	<0.0001
rCBV leakage* Rputamen	rCBF leakage* Rputamen	0.588	<0.0001
rCBV leakage* Rputamen	rCBF leakage* Lthalamus	0.595	<0.0001
rCBV leakage* Rputamen	rCBV leakage* FLAIR	0.604	<0.0001
rCBV leakage* Rputamen	rCBF leakage* FLAIR	0.621	<0.0001
rCBV leakage* Rputamen	rCBF leakage* Rcaudate	0.626	<0.0001
rCBV leakage* Rputamen	rCBF leakage* Lcaudate	0.640	<0.0001
rCBV leakage* Rputamen	rCBF leakage* Rthalamus	0.650	<0.0001
rCBV leakage* Rputamen	rCBF leakage* Lputamen	0.669	<0.0001
rCBV leakage* Rputamen	MTT leakage* active (Gd+) lesions	0.693	0.0029
rCBV leakage* Rputamen	rCBV leakage* active (Gd+) lesions	0.712	0.002
rCBV leakage* Rputamen	rCBV leakage* Rglobus pallidus	0.729	<0.0001

rCBV leakage* Rputamen	rCBV leakage* Lputamen	0.836	<0.0001
rCBV leakage* Rputamen	rCBV leakage* Rcaudate	0.843	<0.0001
rCBV leakage* Rputamen	rCBV leakage* Rthalamus	0.875	<0.0001
rCBV leakage* Rputamen	rCBV leakage* Lthalamus	0.877	<0.0001
rCBV leakage* Rputamen	rCBV leakage* Lcaudate	0.912	<0.0001
rCBV leakage* Rthalamus	rCBF leakage* Lglobus pallidus	0.506	0.0002
rCBV leakage* Rthalamus	rCBF leakage* Rglobus pallidus	0.547	<0.0001
rCBV leakage* Rthalamus	rCBV leakage* FLAIR	0.554	<0.0001
rCBV leakage* Rthalamus	rCBF leakage* Rcaudate	0.556	<0.0001
rCBV leakage* Rthalamus	rCBF leakage* Lcaudate	0.557	<0.0001
rCBV leakage* Rthalamus	rCBF leakage* FLAIR	0.571	<0.0001
rCBV leakage* Rthalamus	rCBF leakage* active (Gd+) lesions	0.579	0.0188
rCBV leakage* Rthalamus	rCBV leakage* Lglobus pallidus	0.598	<0.0001
rCBV leakage* Rthalamus	rCBV leakage* active (Gd+) lesions	0.649	0.0065
rCBV leakage* Rthalamus	rCBF leakage* Lthalamus	0.650	<0.0001
rCBV leakage* Rthalamus	rCBV leakage* Rglobus pallidus	0.666	<0.0001
rCBV leakage* Rthalamus	rCBF leakage* Rthalamus	0.737	<0.0001
rCBV leakage* Rthalamus	rCBV leakage* Rcaudate	0.772	<0.0001
rCBV leakage* Rthalamus	rCBV leakage* Lcaudate	0.814	<0.0001
rCBV leakage* Rthalamus	rCBV leakage* Lthalamus	0.937	<0.0001
volume of active (Gd+) lesions	number of active (Gd+) lesions	0.989	<0.0001
volume of lesions in T1	number of lesions in T1	0.840	<0.0001
Correlations between demographics/clinical characteristics and laboratory biomarkers			
BMI	C3	0.592	<0.0001
Number of relapses in the previous 24 months	Vitamin A	0.512	0.0004
number of relapses in the previous 12 months	Vitamin A	0.540	0.0002
Correlations between demographics/clinical characteristics and radiological metrics			
Age	rCBF leakage* active (Gd+) lesions	0.634	0.0084
Age	rCBV leakage* active (Gd+) lesions	0.704	0.0023
Z_SCORE_T25_Fw	rCBF leakage* active (Gd+) lesions	-0.623	0.0131
Disease duration	rCBV leakage* active (Gd+) lesions	0.619	0.0105
Disease duration	rCBF leakage* active (Gd+) lesions	0.761	0.0006
Number of relapses in the previous 24 months	rCBF leakage* active (Gd+) lesions	-0.549	0.0275
Number of relapses in the previous 12 months	rCBF leakage* active (Gd+) lesions	-0.734	0.0012
Number of relapses in the previous 12 months	rCBV leakage* active (Gd+) lesions	-0.559	0.0244
Z_SCORE_9-HPT	rCBV leakage* active (Gd+) lesions	-0.609	0.0159
Correlations between laboratory biomarkers and radiological metrics			
Chlamydia_IgA	rCBF leakage* active (Gd+) lesions	-0.587	0.0169
Cytomegalovirus_IgG	rCBV leakage* active (Gd+) lesions	0.522	0.0381
Cytomegalovirus_IgG	rCBF leakage* active (Gd+) lesions	0.598	0.0145
Cytomegalovirus_IgM	rCBV leakage* active (Gd+) lesions	0.595	0.0151
Cytomegalovirus_IgM	MTT leakage* active (Gd+) lesions	0.828	0.0001
Epstein-Barr_IgM	MTT leakage* active (Gd+) lesions	0.502	0.0476
RBC	rCBV leakage* active (Gd+) lesions	-0.515	0.0413
Anti-prothrombin__IgM	MTT leakage* active (Gd+) lesions	0.524	0.045
FII	rCBV leakage* active (Gd+) lesions	0.537	0.032
HerpesSimplexType1_IgM	rCBV leakage* active (Gd+) lesions	0.511	0.0432
aC4	rCBF leakage* active (Gd+) lesions	-0.610	0.0157
Tissue_Factor	MTT leakage* active (Gd+) lesions	-0.746	0.0009
P-Selectin\CD62P	MTT leakage* active (Gd+) lesions	-0.645	0.007
P-Selectin\CD62P	rCBV leakage* active (Gd+) lesions	-0.529	0.035
InfluenzaB_IgA	MTT leakage* active (Gd+) lesions	0.532	0.034

InfluenzaB_IgA	rCBF leakage* active (Gd+) lesions	0.607	0.0127
InfluenzaB_IgA	rCBV leakage* active (Gd+) lesions	0.777	0.0004
MHC	rCBV leakage* active (Gd+) lesions	0.535	0.0329
Mycoplasma_IgA	MTT leakage* active (Gd+) lesions	-0.511	0.0429
Mycoplasma_IgG	MTT leakage* active (Gd+) lesions	-0.524	0.0372
PC	rCBF leakage* active (Gd+) lesions	0.510	0.0434
PC	MTT leakage* active (Gd+) lesions	0.518	0.04
PC	rCBV leakage* active (Gd+) lesions	0.665	0.0049
PT	MTT leakage* active (Gd+) lesions	-0.543	0.0299
PT	rCBV leakage* active (Gd+) lesions	-0.533	0.0334
PT%	rCBV leakage* active (Gd+) lesions	0.529	0.0352
PT%	MTT leakage* active (Gd+) lesions	0.548	0.0278
T-CD8	rCBF leakage* active (Gd+) lesions	0.549	0.0342
Vitamin D	FLAIR lesion load(ml)	-0.513	0.0063
Vitamin D	Volume lesions in T1(ml)	-0.504	0.0074
Vitamin K	rCBV leakage* active (Gd+) lesions	-0.827	0.0017
Vitamin K	rCBF leakage* active (Gd+) lesions	-0.720	0.0125
Vitamin K	MTT leakage* active (Gd+) lesions	-0.692	0.0182
vWF	rCBV leakage* active (Gd+) lesions	0.521	0.0462

* A leakage correction analysis was also performed, in order to correct for contrast agent extravasation.
For the abbreviations and units of all laboratory and MRI parameters, see Supplementary Table 1 and Supplementary Table 2.
BMI=body mass index; EDSS=Expanded Disability Status Scale; 9-HPT=9 Hole peg test; MSFC=Multiple Sclerosis Functional Composite.
